# Membrane-bound Heat Shock Protein mHsp70 Is Required for Migration and Invasion of Brain Tumors

**DOI:** 10.1158/2767-9764.CRC-24-0094

**Published:** 2024-08-12

**Authors:** Maxim Shevtsov, Danila Bobkov, Natalia Yudintceva, Ruslana Likhomanova, Alexander Kim, Evegeniy Fedorov, Viacheslav Fedorov, Natalia Mikhailova, Elena Oganesyan, Sergey Shabelnikov, Oleg Rozanov, Timur Garaev, Nikolay Aksenov, Alla Shatrova, Artem Ten, Anastasiya Nechaeva, Daria Goncharova, Rustam Ziganshin, Anastasiya Lukacheva, Daria Sitovskaya, Alexey Ulitin, Emil Pitkin, Konstantin Samochernykh, Evgeny Shlyakhto, Stephanie E. Combs

**Affiliations:** 1 Klinikum Rechts der Isar, Technical University of Munich, Munich, Germany.; 2 Personalized Medicine Centre, Almazov National Medical Research Centre, St. Petersburg, Russia.; 3 Institute of Cytology of the Russian Academy of Sciences (RAS), St. Petersburg, Russia.; 4 School of Medicine and Life Sciences, Far Eastern Federal University, Vladivostok, Russia.; 5 Smorodintsev Research Institute of Influenza, St. Petersburg, Russia.; 6 Shemyakin-Ovchinnikov Institute of Bioorganic Chemistry Russian Academy of Sciences (RAS), Moscow, Russia.; 7 Polenov Neurosurgical Institute, Almazov National Medical Research Centre, St. Petersburg, Russia.; 8 Wharton School, University of Pennsylvania, Philadelphia, Pennsylvania.

## Abstract

Molecular chaperones, especially 70 kDa heat shock protein, in addition to their intracellular localization in cancer cells, can be exposed on the surface of the plasma membrane. We report that the membrane-associated chaperone mHsp70 of malignant brain tumors is required for high migratory and invasive activity of cancer cells. Live-cell inverted confocal microscopy of tumor samples from adult (*n* = 23) and pediatric (*n* = 9) neurooncologic patients showed pronounced protein expression on the membrane, especially in the perifocal zone. Mass spectrometry analysis of lipid rafts isolated from tumor cells confirmed the presence of the protein in the chaperone cluster (including representatives of other families, such as Hsp70, Hsc70, Hsp105, and Hsp90), which in turn, during interactome analysis, was associated with proteins involved in cell migration (e.g., Rac1, RhoC, and myosin-9). The use of small-molecule inhibitors of HSP70 (PES and JG98) led to a substantial decrease in the invasive potential of cells isolated from a tumor sample of patients, which indicates the role of the chaperone in invasion. Moreover, the use of HSP70 inhibitors in animal models of orthotopic brain tumors significantly delayed tumor progression, which was accompanied by an increase in overall survival. Data demonstrate that chaperone inhibitors, particularly JG98, disrupt the function of mHsp70, thereby providing an opportunity to better understand the diverse functions of this protein and offer aid in the development of novel cancer therapies.

**Significance:**

Membrane-bound mHsp70 is required for brain tumor cell migration and invasion and therefore could be employed as a target for anticancer therapies.

## Introduction

Molecular chaperones (HSPs), especially Hsp70, play an important role in maintaining cellular homeostasis by regulating the assembly of polypeptide chains, the organization of tertiary and quaternary protein structures, participation in the processes of apoptosis and autophagy (1–4). Therefore, in many types of tumor cells there is an increased expression of chaperones, which leads to an increase in the stress resistance of cells to the effects of radio- and chemo-therapies, the effects of a hostile tumor microenvironment (e.g., oxidative stress, hypoxia, and nutrient deprivation; refs. 5–7). Apart from their intracellular localization, it was shown that certain members of the HSP family (e.g., HSP40, HSP60, HSP70, and HSP90) could be localized on the surface of the plasma membrane of tumor cells but not on corresponding normal cells (8, 9). Although among the described membrane-associated chaperones, the Hsp70 protein has been most often studied as a target for the development of new diagnostic and therapeutic (i.e., theranostic) agents in oncology, its function is still poorly understood (10, 11). Several functions were attributed to the oligomerized HSP70 upon plasma membrane insertion including the formation of the ion conductance channels (selective for cations and not voltage-dependent; ref. 12), membrane stabilization and induction of quasiinterdigitated lipid phase (13–16), endocytosis (17), participation in extracellular export mechanism (18), and signal transduction (19). Although no regions have been found in the chaperone’s structure that could explain its anchoring in the cell plasma membrane, a number of studies have suggested its interaction with certain lipids through electrostatic and hydrophobic interactions, including phosphatidylserine (16, 20, 21) and glycosphingolipid globotriaosylceramide (Gb3) for lipid rafts (22, 23).

Recent reports have indicated that Hsp70 might also be involved in the tumor cell invasion and metastasis (24–29) and motility of normal cells (30–32). Thus, in the studies by Jagadish and colleagues (25, 26) it was shown that the knockdown of the Hsp70 by shRNA substantially inhibited invasive (more than 60% of inhibition) and migratory abilities of CRC cells (i.e., COLO205, HCT116) and reduced motility of the BC cells (i.e., MDA-MB231, and MCF7). This effect in terms of cell motility was further confirmed by Gupta and colleagues (27) in epithelial ovarian cancer cells. In another study by Liu and colleagues (33), it was demonstrated that overexpression of 14-3-3σ isoform (stratifin) induced the expression of heat shock factor-1α (HSF1α) and Hsp70 in hepatocellular carcinoma cells that in turn increased the tumor cell motility (but decreased cell invasion) mediated by the GSK3β/β-catenin signaling pathway. In a similar work downregulation of Hsp70 by the chaperone inhibitors (VER155008 and YM08) dramatically suppressed the TGFα-induced migration of human hepatocellular carcinoma–derived HuH7 cells (via AKT signaling pathway; ref. 28). However, in the forementioned studies the authors did not differentiate between cytosolic, nuclear, and membrane-bound forms of the Hsp70 (usually employing fixed and permeabilized either cells or tissue specimens for the detection of chaperone), predominantly focusing on the role of the Hsp70 in mediating the intracellular signaling pathways involved in cell motility and cell cycle. Boroughs and colleagues (34) in their studies had come closest to the possible involvement of a membrane-bound Hsp70 in the invasion of cancer cells when in their observation employing microscopy studies, authors demonstrated an association of the protein with the tTG translocated to the plasma membrane.

In the current study, we evaluated the expression of the membrane-bound mHsp70 in brain tumors and brain metastasis, particularly in glioblastomas, employing live-cell inverted confocal microscopy of the intraoperatively obtained material from neurooncologic patients. We could demonstrate the notable expression of mHsp70 in cancer cells, particularly in the peritumoral zone, that was associated with increased cell motility and invasive potential (as proved by *in vitro* studies). Indeed, it is the invasive properties of cells that determine the diffuse spread of gliomas into the surrounding normal brain tissue, which ensures a high percentage of tumor recurrence (35). Subsequent experiments with the application of mHsp70 inhibitors *in vivo* further demonstrated the therapeutic potency (substantially reducing the tumor growth as shown by MRI studies) of the membrane-bound chaperone as a plausible target for the treatment of malignant brain tumors.

## Materials and Methods

### Patients

The expression of mHsp70 was evaluated by inverted live-cell confocal microscopy of the intraoperative biopsy material obtained from newly diagnosed untreated neurooncologic patients (*n* = 23) from December 2020 to April 2024 (Table 1). The study protocol was conducted in accordance with the Declaration of Helsinki and was approved by the Ethics Committee of the Almazov Medical Research Centre (approval No. 2712-20, December 21, 2020). All patients gave their informed written consent to be included in the study. We confirm that all experiments were performed in accordance with the relevant guidelines and regulations. The study included patients with a radiologic diagnosis of malignant glioma: tumors with a distinctive ring-enhancing pattern with thick irregular walls on MRI and a core area suggesting tumor necrosis. Exclusion criteria were midline, basal ganglia, or brain stem tumors as assessed by MRI.

**Table 1:** Patients and tumor characteristics

Variables	Number (%)
Patients with adult brain tumor	
Number of patients	23 (100%)
Age (years)	60 (range 47–64)
Sex	
Male	16 (66.7%)
Female	7 (33.3%)
Karnofsky performance scale before surgery	70 (range 60–70)
Dexamethasone before surgery (mg)	12 (range 12–16)
Tumor site (lobe)	
Frontal	6 (26.1%)
Temporal	5 (21.7%)
Parietal	1 (4.3%)
Frontoparietal	3 (13.1%)
Frontotemporal	1 (4.3%)
Parietooccipital	3 (13.1%)
Occipitotemporal	1 (4.3%)
Multiple	3 (13.1%)
Hemisphere	
Right	16 (69.6%)
Left	7 (30.4%)
Histology	
Glioblastoma, idh-wildtype, grade 4	19 (82.7%)
Anaplastic oligodendroglioma, idh-mutant and 1p/19q-codeleted, grade 3	2 (8.7%)
Astrocytoma, idh- mutant, grade 3	1 (4.3%)
Astrocytoma, idh- mutant, grade 3	1 (4.3%)
Mib1 index (%)	20 (range 15–30)
Fluorescence-guided surgery with 5-aminolevulinic acid	
No	19 (82.7%)
Yes	4 (17.3%)
Patients with pediatric brain tumor	
Number of patients	9 (100%)
Age (years)	8 (range 3–11)
Sex	
Male	6 (66.6%)
Female	3 (33.3%)
Lansky scale before surgery	70 (range 50–90)
Dexamethasone before surgery (mg)	5 (range 2–8)
Tumor site	
Thalamus	4 (44.4%)
Hemysfer	3 (33.3%)
Pons	1 (11.1%)
Septum	1 (11.1%)
Histology	
Glioblastoma IDH-wildtype, grade 4	3 (33.3%)
Diffuse midline glioma H3 K27-altered, grade 4	4 (44.4%)
Diffuse pediatric-type high-grade glioma H3-wildtype and IDH-wildtype, grade 4	1 (11.1%)
Pleomorphic xanthoastrocytoma, grade 3	1 (11.1%)
Fluorescence-guided surgery with 5-aminolevulinic acid	
No	6 (66.6%)
Yes	3 (33.3%)

All imaging was performed using the 3T MR scanner (MAGNETOM Skyra 3,0T, Siemens, Germany) applying a standardized MRI, 1H-MRS, DWI, DTI, and DSCE examination patient protocol with a 4-channel birdcage and an 8-channel phased-array head coil. Standard anatomic MRI sequences included precontrast sagittal, transverse, and coronal T2-weighted FSE [repetition time (TR)/echo time (TE) 4,800 ms/103 ms, and slice thickness 3 mm], precontrast axial T2-weighted fluid attenuation inversion recovery (FLAIR; TR/TE 9,502 ms/126 ms, slice thickness: 5 mm), pre- and post-contrast axial T1-weighted FSE images (TR/TE 700 ms/9.4 ms, slice thickness: 5 mm) were also obtained, postcontrast isotropic 3-dimensional spoiled gradient echo [3D-SPGR, TR/TE 6.8 ms/2.1 ms, 12° flip angle, 240 × 240 mm^2^ field of view (FOV), 136 slices, slice thickness: 1.2 mm].

In all cases, tumor resection was performed according to the concept of information-guided surgery based on the integrated analysis of various intraoperative data reflecting anatomical, functional, and histopathologic features of the clinical case, which presumes routine use of frameless neuronavigation system (Radionics OmniSight, USA), ultrasound neuronavigation (ultrasonic scanner Sonoline Siemens, Germany), comprehensive intraoperative neurophysiologic monitoring (NicoletOne, USA; Inomed, Germany) were used, and histopathologic monitoring of the resected tissue. Fluorescence-guided surgery with 5-aminolevulinic acid (Alasense, Federal State Unitary Enterprise “SSC” “NIOPIK,” Russia) was performed in some patients with a Karnofsky performance scale of 60 and more, no renal insufficiency (i.e., creatinine < 177 μmol/L), and no hepatic insufficiency (i.e., gamma-glutamyl transpeptidase < 100 U/L). Patients received freshly prepared solutions of 5-aminolevulinic acid (20-mg/kg body weight) orally for 3 hours (range, 2–4) prior to induction of anesthesia. Solutions were prepared by dissolving the contents of a vial (1.5 g) in 50 mL of drinking water. The operating microscope (Pentero 700, Carl Zeiss Meditec AG, Germany; Leica M500N OHS1, Leica M720 OH5 Germany) was switched to violet–blue illumination for fluorescence visualization as requested by the surgeon.

Samples of histologic material were obtained from three tumor regions according to preoperative planning based on MR scanning (36): (i) necrotic zone (the tumor center presented in unenhanced on T1-weighted images), (ii) tumor contrast enhancement area of the tumor on T1-weighted images, and (iii) peritumoral region 5 mm from the enhancement margin on the hyperintense FLAIR scanning. Histopathologic tumor typing and grading were based on the standard criteria of the fifth edition of the World Health Organization classification of central nervous system tumors (2021; ref. 37). Initially, a macroscopic examination of the biopsy material was carried out. Tumor samples without visible signs of necrosis were subsequently immersed in formalin solution (10%) and fixed in it for a day (temperature 18°C–20°C). The tumor fragments were then treated in distilled water for 2 hours, then in increasing strength alcohols, and fixed in paraffin blocks. Using a microtome, paraffin sections were made, in which thickness was approximately 4 to 6 μm, which were applied to glass slides with heated strong alcohol. For 1 to 2 minutes, the section was treated in a xylene solution for deparaffinization. For washing off paraffin preparation was washed first in 96% ethanol, then with distilled water, after which it was treated with a solution of hematoxylin for 5 to 10 minutes and with a solution of eosin for 1 minute, followed by retreatment with 96% ethanol for 5 to 10 minutes and distilled water. After removing the alcohol residues, the preparation was dried, xylene (an antireflection agent) was applied to it, and the section was fixed under a coverslip with a drop of Canadian balsam. Formalin fixed–paraffin embedded tissues were obtained and submitted for immunostaining with an automated stainer (Lab Vision Autostainer 360 ThermoScientific). The following primary antibodies were used: GFAP (polyclonal; DakoCytomation, Denmark), Synaptophysin (clone 27G12; DakoCytomation, Denmark), Olig2 (clone OLIG2, Abcam, UK), Ki67 (clone MIB1; DakoCytomation, Denmark), IDH 1r132h (clone H09; Dianova Int., Germany), MGMT (clone MT3.1; Diagnostic BioSystems, USA), ATRX (clone D5; Diagnostic BioSystems, USA), and EGFR (clone 31G7; Diagnostic BioSystems, USA). In tumors with oligodendroglial morphology, 1p/19q status was assessed using FISH.

The study also included intraoperative biopsy of pediatric neurooncologic patients with a radiological diagnosis of malignant glioma (*n* = 9), for the same period of time (Table 1). The study protocol was approved by the Ethics Committee of the Almazov Medical Research Centre (Approval No. 0210-22, October 31, 2022).

MRI of the brain was performed on expert-class tomograph Magnetom Espree (Siemens, Germany) with a magnetic field induction of 1.5 T, including pulse sequences T1, T2, FLAIR, and T1 contrast (which meets the criteria of RANO). The thickness of the slices constituted 1 to 3 mm. For the evaluation of mHsp70 expression in the nontumorous brain tissues, we employed patient-derived samples obtained intraoperatively from patients with drug-resistant epilepsy who underwent temporal resection (*n* = 5).

### Live-confocal microscopy of tumor samples

During surgery, small (50–100 mm^3^) pieces of tissue were removed from patients. The resulting material was washed three times in a PBS solution at room temperature, gently shaking for 10 minutes, and stained for 1 hour with a mixture of TMRM (tetramethylrodamine methyl ester) dyes (1 μmol/L), Hoechst 33342 (0.1 μg/mL), and monoclonal antibodies against Hsp70 [SPA 810 (StressMarq)] conjugated with FITC, to identify the membrane potential of mitochondria, DNA, and membrane-bound Hsp70, respectively. After staining, the material was washed with PBS and placed in a thin-bottomed Ibidi μ-Dish 35-mm dish and covered with a cover glass to ensure uniform adherence of the sample to the surface of the bottom of the dish. The material was then examined by confocal laser scanning microscopy using a Leica TCS SP8 (Leica Microsystems, Germany) inverted microscope equipped with argon and helium–neon lasers. Hoechst 33342 fluorescence was excited with a 405 nm laser and recorded in the range 415 to 500 nm, Hsp70-FITC fluorescence was excited with a 488 nm laser and recorded in the range 495 to 560 nm, TMRM fluorescence was excited with a 561-nm laser and recorded in the range 565 to 700 nm. Separate scanning was carried out in three channels, and the width of the confocal aperture was set to 100 μm, which approximately corresponds to the thickness of the optical section of 1 μm. An HC PL APO 63×/1.40 OIL CS2 oil immersion lens was used. Images were obtained at a resolution of 1,024 × 1,024 pixels with an average of three along each scanning line. Throughout the experiment, the same laser power and detector voltage settings were used to obtain all images. Images obtained from unstained material were used as a control for autofluorescence. At least 10 images were obtained from each sample, and the average intensities of green (Hsp70) pixels were calculated from confocal images. To do this, the background was subtracted from each image using ImageJ (38), using the Rolling boll algorithm with a parameter of 50 pixels. The images were then processed in the free software environment R, in which average pixel intensities were extracted using the EBImage library (39).

### Isolation of lipid rafts

Membrane rafts were prepared from samples by nonionic detergent lysis and discontinuous sucrose gradient centrifugation. The tissue sample was placed in a Dounce homogenizer and lysed for 15 minutes on ice in 400 μL of lysis buffer (100-mmol/L NaF, 50-mmol/L KCl, 2-mmol/L MgCl2, 1-mmol/L egtazic acid, 0.5-mol/L sucrose, 1% Triton X100, and 10-mmol/L potassium phosphate buffer pH 7.0) with Complete Protease Inhibitor Cocktail (Roche Applied Science). Lysates were then homogenized by passing them through a 22-gauge needle 20 times before being centrifuged at 10,000 rpm for 10 minutes (+4°C). The bottoms of centrifuge tubes were filled with supernatant solutions that had been gently mixed with 1 mL of 60% OptiPrep (Serumwerk Bernburg AG, Norway). A series of OptiPrep dilutions (50%, 40%, 35%, and 0%) were prepared in lysis buffer.

These were layered on top of the sample in order, with the highest concentration starting at the bottom, to form the initial gradients; these were layered on top of the sample in the order listed, with the highest concentration at the bottom. The samples were spun at 200,000 *g* in an Optima XPN90 ultracentrifuge (Beckman Coulter, Germany) at 4°C for 20 hours. After centrifugation, 10 fractions (0.5 mL each) were collected from above, fractions 3 to 5 were pooled and kept at −20°C.

### Liquid chromatography and mass spectrometry

#### Sample preparation

Proteins were precipitated by adding 10 μL of 1% sodium deoxycholate solution to 500 mL of sample, followed by acidification with 50 μL of trichloroacetic acid. The resulting suspension was precipitated by centrifugation, washed with acetone, and dried. The precipitate was dissolved in 8-mol/L urea and treated sequentially with dithiothriethol and iodoacetamide. The reaction mixture was diluted 10 times with 50-mmol/L Tris buffer, pH = 8.0, and 1 μg of trypsin (Trypsin Gold, #V5280, Promega) was added. The trypsinolysis reaction was carried out for 18 hours at 37°C. Peptides were isolated using solid phase extraction on reverse-phase cartridges (30 mg, Strata-X, #8B-S100-TAK, Phenomenex) and concentrated using a rotary vacuum evaporator. Before drying, 10 μL of 50-mg/mL D-glucose solution was added to the peptides to facilitate subsequent solubilization. The dry residue was dissolved in 50 μL of 1% formic acid and filtered through a 0.2-μm polyvinylidene difluoride filter.

#### HPLC-MALDI analysis

The resulting peptide mixture was separated on a Chromolith CapRod RP18e HR reverse-phase column (0.1 × 150 mm^2^, Merck) using a water-acetonitrile gradient on an Eksigent NanoLC Ultra 2D+ system (SCIEX) nano-HPLC system. The eluate was fractionated and applied to a MALDI target using a robotic microfraction collector. Each chromatogram was divided into 1,408 fractions; the target contained two chromatograms. Fractionated samples were analyzed on a TOF/TOF 5800 System (SCIEX) time-of-flight tandem MALDI mass spectrometer in positive ion mode. Spectra of parent ions were obtained at a laser intensity of 3,000 U and 1,000 shots per spectrum. For fragmentation, up to 25 parent ions were automatically selected from each point in the mass range 750 to 3,500 Da and the signal-to-noise ratio of at least 40. Fragmentation spectra were obtained at a laser intensity of 3,800 U and up to 1,500 shots per spectrum with the DynamicExit algorithm enabled.

### Data analysis

MS raw files were analyzed by Peaks studio 10.0 (Bioinformatics Solutions Inc.; ref. 40). Identification of proteins was made by searching against the *Homo sapiens* Uniprot FASTA database version of 09.07.2021 with a carbamidomethyl Cys as a fixed modification and deamidation Asn/Gln and Met oxidation as variable modifications. FDR for peptide-spectrum matches was determined by searching a reverse database and was set to 0.01. Enzyme specificity was set as C-terminal to arginine and lysine, and a maximum of two missed cleavages were allowed in the database search. Peptide identification was performed with an allowed initial precursor mass deviation of up to 10 ppm and an allowed fragment mass deviation of 0.05 Da. To calculate the protein abundance index, the total number of peptides identified for a protein was divided by the predicted length of this protein.

The list of proteins identified by MS was loaded into the STRING multiple proteins search online tool (41) with the following settings to generate a network of protein–protein interactions (PPI): organism—*Homo sapiens*; network type—full STRING network; meaning of network edges—evidence; and minimum required interaction score—medium confidence (0.4). The resulting network of protein–protein interactions was obtained from the proteome using the STRING database and then clustered using the Markov cluster algorithm (42) with an inflation parameter set to four to identify protein complexes. In the resulting clusters, filtering by compartment (the association parameter with the plasma membrane is greater than or equal to 4) identified proteins strongly associated with the plasma membrane. To visually represent the variation in node degrees and highlight the interactomes for the obtained protein clusters, the STRING PPI network was imported into the Gephi program (43) with the following settings: ForceAtlas 2 layout with no node overlap allowed; node size ranking based on degree with a size range of 10 to 500. To extract nonoverlapping communities from the STRING PPI network the Louvain method was used (44).

### Multiplex analysis of tumor samples

The postoperative material was fixed in a 10% formalin solution (pH = 7.0). Histologic preparations were prepared in accordance with the standard protocol. Sections with a thickness of 1.5 to 2 μm were made using a rotary microtome HM 325 (Thermo Fisher Scientific, USA). The preparations were deparaffinized and antigens were unmasked. Then the preparations were stained using an Opal 3-Plex Manual Detection Kit (Akoya Biosciences, United States). The preparations were sequentially incubated with primary antibodies: anti-Hsp70 (Abcam, USA), antinestin (Santa Cruz, USA), and anti-SOX2 (Chemicon, USA) for 30 minutes. Dilution of primary antibodies was performed in a ratio of 1:100. Universal antibodies with a polymer conjugated with horseradish peroxidase (Opal Polymer Antirabbit HRP Kit) were used as secondary, incubation with which was performed for 30 minutes. The preparations were washed three times with PBS solution and Opal (fluorochrome) was applied for 10 minutes. Each primary antibody (anti-Hsp70, antinestin, and anti-SOX2) was assigned a specific fluorochrome (Opal 520, Opal 570, and Opal 690, respectively). After incubation with Opal 520 fluorochrome (to remove antibodies and retain only fluorochrome), the slides were placed in an EDTA solution (versene) and heated for 20 minutes in a water bath at 98.5°C. Processing was performed sequentially for each fluorochrome. At the last stage, cell nuclei were stained with 4′,6-diamidino-2-phenylindole (DAPI; Abcam, United Kingdom) and preparations were mounted in a Mounting medium (Abcam, United Kingdom).

Stained preparations were analyzed using a Mantra 2 Quantitative Pathology Workstation confocal microscopy system equipped with the Pathology Views software for conventional brightfield fluorescence image analysis. Nuclei were detected using a diode laser (405 nm) and fluorochromes (Opal 520, Opal 570, and Opal 690) using lasers with the appropriate wavelength.

To quantify the colocalization of Hsp70 with the nestin and SOX2 the phenotyping map was constructed. According to the staining of DAPI nuclei isolated cell nuclei were identified. Cells containing Hsp70, nestin, and SOX2 were marked with red, magenta, and green colors, respectively. The preparation was conditionally segmented into a necrosis area (blue color) and a zone of the surrounding viable tissue (VT; brown color) and holes (green color). Identification of markers and evaluation of the localization of two markers is expressed in %. Cells (with and without markers) were counted in the entire image and taken as 100%. Based on a phenotyping map using the Pathology Views fluorescence image analysis software, Hsp70 colocalization was quantified with nestin and SOX2 in the region of the VT and necrosis area.

### Cells

Tumor cell lines C6 (rat glioma) and T98G (human glioblastoma) were provided by the Russian Cell Culture Collection (Institute of Cytology, Russian Academy of Sciences, St. Petersburg, Russia). Tumor cell line U251 (human glioblastoma) was provided by the N.N. Petrov National Medicine Research Center of oncology of the Ministry of Health of the Russian Federation (St. Petersburg, Russia). Cells were cultured in DMEM/F12 medium (Gibco, USA) supplemented with 10% fetal bovine serum (FBS) (Gibco, USA) and gentamicin (Gibco, USA) at a working concentration of 50 μg/mL at 37°C in a 5% CO_2_ incubator. For experiments, single-cell suspension was obtained using 0.25% trypsin-EDTA (Gibco, USA). All cell lines were tested for the expression of mHsp70 by employing flow cytometry and confocal microscopy.

Primary glioblastoma cells were isolated from biopsy material obtained during glioblastoma surgical removal and cultured (45). Cells were grown in DMEM/F12 medium (Gibco, USA) supplemented with 0.5% 1-mol/L 4-(2-hydroxyethyl)-1-piperazineethanesulfonic acid buffer (Sigma-Aldrich, USA), 1% B27 Supplement minus vitamin A (Thermo Fisher Scientific, USA), and recombinant human EGF and bFGF (SCI store, Russia) at a working concentration of 20 ng/mL. All cells were examined for mHsp70, SOX2, and nestin expression by employing confocal microscopy. Small pieces of tumors (0.5 mm^3^) were also cultivated in the form of a 3D explant model at 37°C in a 5% CO_2_ incubator. After several months, migrated cells spontaneously formed spheroids. The obtained suspension culture of primary glioblastoma cells was shifted to an adhesive state by gradually changing the tumor sphere culture medium to DMEM/F12 medium supplemented with 10% FBS.

### Confocal microscopy

The expression of mHsp70 in tumor cells was visualized by the confocal system Olympus FV3000 (Olympus, Japan). C6, T98G, and U251 cells (0.1 × 10^6^ cells mL^−1^) were allowed to settle on glass slides. Cells were incubated with FITC-conjugated anti-Hsp70 monoclonal antibody (mAb) against membrane-bound Hsp70 SPA810 (StressMarq) for 30 minutes on ice. Following incubation, cells were washed with PBS and fixed in a 10% formalin solution (Sigma-Aldrich, USA). Glasses were mounted in Ibidi Mounting Medium with DAPI (Ibidi, Germany). Сells incubated with FITC-conjugated isotype-matched (IgG1) control antibody and unstained cells were used as controls. To quantify the mean signal intensity of mHsp70 on the plasma membrane of C6, T98G, and U251 cell lines, confocal images were examined using the ImageJ program (*n* ≥ 20). Statistical analysis of the results was performed using the GraphPad Prism 8 program (GraphPad Software, USA). Primary glioblastoma cells were also stained with FITC-SPA810 mAb, eFluor 570-SOX2 mAb (Thermo Fisher Scientific, USA), and Alexa Fluor 647-nestin mAb (BioLegend, USA).

### Flow cytometry and cell sorting

Analysis of mHsp70 expression was performed by the CytoFLEX flow cytometer (Beckman Coulter, USA) using a FITC-SPA810 antibody. Briefly, the suspension of tumor cells (0.3 × 10^6^ cells mL^−1^) was incubated for 30 minutes on ice with FITC-SPA810 and then washed with cold PBS. Сells incubated with FITC-IgG1 and unstained cells were used as controls. Based on the cytograms, each cell line was sorted into two subpopulations—high expressed mHsp70 (mHsp70^High^) and low expressed mHsp70 (mHsp70^Low^), using the FITC-anti-Hsp70 antibody. FACS was performed on S3e Cell Sorter (Bio-Rad, USA) and the 488 nm laser. Selected mHsp70^High^ cells C6, T98G, and U251 accounted for 3%, 8%, and 4% of the total population, respectively. Selected mHsp70^Low^ cells C6, T98G, and U251 accounted for 34%, 25%, and 20% of the total population, respectively. mHsp70^High^ cells had a fluorescence intensity 10-fold higher than mHsp70^Low^ cells. The gating of cells using FACS is shown in Supplementary Fig. S1. Additionally, cells were stained by PE-anti-CD133 antibodies (Abcam, USA). CD133 staining of C6, T98G, and U251 cell lines revealed that C6 and T98G cells have no CD133 protein on their surface (CD133^−^; Supplementary Fig. S2). However, the U251 line had the opposite result, 100% of the cell population stained with CD133 antibodies (CD133^+^). All subsequent migration experiments with cell lines were performed at the first passage after FACS. To assess the effect of 2-phenylethynesulfonamide/pifithrin-μ (PES; Sigma-Aldrich, USA) and benzothiazole rhodacyanine (JG98; MedChemExpress, USA) inhibitors on mHsp70 expression, C6, T98G, and U251 cells were preincubated with PES (C = 1 mmol/L) and JG98 (C = 50 nmol/L) for 24 hours, after which samples were prepared for flow cytometry as described above.

### Wound healing assay

Cell subpopulations with high and low expression of mHsp70 were seeded into a two-well silicone insert in a 35-mm dish (Ibidi, Germany). Cells were cultured until monolayer confluence was reached. Subsequently, scratch wounds were generated by removing the barrier insert from the dish. Dishes were washed with a culture medium to remove floating cells. Wounds were imaged and analyzed by CellVoyager CQ1 Benchtop High-content Analysis System (Yokogawa, Japan) for 30 to 48 hours depending on the rate of cell migration. The resulting graphs were performed using the Prism GraphPad 8 program.

### Manual cell motility analysis

The motility characteristics of tumor cells were studied using long-term live-cell imaging. The day before the experiment, cells were seeded with ∼10% confluency in 6×/12×/24× plates. No additional cell manipulations were performed in the 3D explant model. 0.1% Hoechst 33342 (Invitrogen, USA) was added to the cell medium 30 minutes before the test, and, optionally, Hsp70 inhibitors were added 5 minutes before the test. Cells were imaged using a CellVoyager CQ1 Benchtop High-content Analysis System (Yokogawa, Japan). Images of the single-cell movement were obtained more than a 24-hour period with an interval of 15 minutes using 405- and 488-nm lasers, brightfield illumination, and a dry 10× objective. All images were 2,528 × 2,136 pixels, in which one pixel represented a signal from 0.6525 μm. For subsequent image processing, the Manual Tracking plugin of the ImageJ program was used. To register the cell track, the X/Y position of the individual cell nucleus was marked manually at each time point (*n* ≥ 50 in each experimental group). Tracks were recorded only for those cells that fit the following conditions: The cell must be in the FOV in all frames, and it must not undergo cell mitosis or apoptosis. Cell trajectory analysis was carried out in the R software environment version 4.0.2. using the trajr package created for the numerical characterization and analysis of the trajectory of moving animals, as well as for cell motility (46, 47). Trajectory analysis allowed us to calculate such cell motility characteristics as mean speed, max speed, straightness, sinuosity of track, and mean square displacement. Using the trajr package, trajectories were simulated using a theoretical correlated random walk model in which the rotation angle of each step is equal to the direction of the previous step ± some error. We employed staggered analysis to show how the mean cell speed changed over time (48). Statistical processing and obtaining graphs were performed using the Prism GraphPad 8 program. The data were first tested for normal distribution by the Shapiro–Wilk and Kolmogorov–Smirnov tests. For the distribution of any differences in groups of data, ANOVA was performed for normally distributed data or the Kruskal–Wallis test for nonnormally distributed data. If the data passed the test for normality, then the Student *t* test (*t* test) was used to identify statistically significant differences. The width of confidence intervals was estimated by the Wilson method for each data set separately. If the data did not pass the *W* test, then the nonparametric Wilcoxon method or the nonparametric Kruskal–Wallis method was used (49, 50). Statistical analysis specifics are presented in the Supplementary Information.

### Automatic cell motility analysis

The cell speed determination of the primary culture of GBM was carried out using the automatic imaging system of cells Image ExFluorer (LCI, Korea). The cells were seeded into 24-well plates, and the cell nuclei were stained with Hoechst 33342 fluorescent dye; after that, the plates were placed into an integrated incubator with temperature maintenance (37°C) and CO_2_ level (5%). Fluorescence was excited at 405 nm and a long-focus semiapochromatic lens (S plan Fluor ELWD) 20× with a numerical aperture of 0.45 was used.

For each cell line, nine wells with five visual fields in each were used: three nontreated wells on different matrices (poly-L-lysine, Matrigel, and fibronectin), three wells with PES inhibitor on matrices, and three wells with JG98 inhibitor on matrices. The FOVs were taken every 15 minutes for 24 hours, and images with a resolution of 2,560 × 2,160 pixels (0.33 μm/px) were obtained with automatic focusing and focus correction in real time. The captured frames were analyzed using the NIS-Elements software with a module for automatic segmentation, quantification, and tracking of individual cells: cell nuclei (with a diameter of more than 2.88 μm) were recognized in images and the *X*–*Y* coordinates of the nuclei were recorded, cell movement tracks were built according to the coordinates of the nuclei, the resulting tracks were filtered by duration (more than 48 hours), and the average speed of movement for 24 hours was calculated.

Thus, tracks were obtained from all cells in the FOV: (i) cells that are part of spheroids and (ii) single cells outside of spheroids. At the same time, only the cells of the second group were assigned to the population of highly invasive cells. To register the tracks of highly invasive cells, we trained an artifical intelligence (AI) model based on the NIS-Elements Advanced Research software to recognize only actively moving cells located outside the spheroids. The model was trained as follows: 10 images were selected from different FOVs, regions of interest around all cells were manually selected on each image, and binary masks were obtained from the selected regions of interest, which, together with the original images, were transmitted to the NIS software module.Ai, in which 1,000 iterations of training were performed. The resulting model was used for the recognition of highly invasive cells and their tracking.

The data obtained were statistically processed using GraphPad Prism software 9.4.1.681 (GraphPad Software Inc., USA). The data are presented as a median with 95% confidence intervals. The normality test was performed using Kolmogorov–Smirnov and Shapiro–Wilk tests. All the data was distributed abnormally. The Mann–Whitney *t* test was used to analyze the differences compared with the control group. The differences were considered statistically significant at *P* < 0.05. Statistical analysis specifics are presented in the Supplementary Information.

### Animals

Male Wistar rats (weighing 280–300 g) were purchased from the animal nursery “Rappolovo” of the Russian Academy of Medical Sciences (RAMN; Rappolovo, Russia). Male C57Bl/6 mice and SCID mice (8–10 weeks old) were obtained from the animal nursery Pushchino (Pushchino, Russia). All rats and mice were kept and bred under specific pathogen-free conditions in accordance with the guidelines of the Federation of European Laboratory Science Association. All animal experiments were performed in compliance with the European Union and approved by the ethical committee of Almazov National Medical Research Centre.

### Orthotopic glioblastoma models, tumor volumetrics, and tumor irradiation

For anesthesia of rats, a mixture of ketamine (87 mg/kg) and xylazine (13 mg/kg) was employed. Animals were fixed into stereotactic frames (David Kopf Instruments, Tujundra, CA). Suspension of C6 cells (1 × 10^6^ cells) in 10 μL of culture medium was injected into *nucl. caudatus dexter*. SCID mice or C57Bl/6 mice were anesthetized with an intraperitoneal injection of fentanyl (0.05 mg/kg), midazolam (5 mg/kg), and medetomidine (0.5 mg/kg) mixture and further fixed in a stereotactic frame. Either U251 or T98G cells (6 × 10^4^ cells) were inoculated at 2.0 mm lateral and 1.2 mm posterior to the bregma (3.0-mm depth).

Tumor progression was evaluated before and after each therapy on days 7, 14, 21, and 30 employing a high-field (11.0T) MR scanner (Bruker, Bremen, Germany, with a customized coil; ref. 51). High-resolution anatomical T_2_-weighted and T_1_-weighted scans in coronal planes were performed. The obtained images were analyzed using software (AnalyzeDirect Inc, Overland Park, KS, USA).

The stereotactic irradiation of the glioma tumors was performed employing X-ray beams on the Small Animal Radiation Research Platform (SARRP; XSTRAHL Ltd., Camberley, UK). Following anesthesia, tumor irradiation was performed using a 225-kV (peak) X-ray beam at a dose rate of 2.5 Gy minutes^−1^ (with a collimator 5.0 × 5.0 mm^2^). The single-dose tumor irradiation was 10 Gy.

### Statistics

The comparative overall survival of the glioma-bearing animals was evaluated with Kaplan–Meier curves that were based on the Kaplan–Meier estimator. When comparing group means of two continuous normally distributed variables, the parametric Student test was used; for nonnormally distributed variables, the Man–Whitney test was used. The significance level for all tests was *α* = 0.05, and all confidence intervals were reported at the 95% level. All *P* values reported for all I-tests were two-sided. When comparing multiple groups as a nonparametric analog to the one-way ANOVA test the Kruskal–Wallis test was employed. Statistica Version 9.2 for Windows or the R programming language was used for all tests. The Benjamini–Yekutieli procedure was used *post hoc* to control multiple comparison (52), and the results were visualized using the Python programming language with the scikit-posthocs package (53).

### Data availability

The datasets used and/or analyzed during the current study are available from the corresponding author Prof. Dr. Maxim Shevtsov upon reasonable request.

## Results

### mHsp70 is overexpressed in brain tumors but not in normal tissues

**Figure 1:**
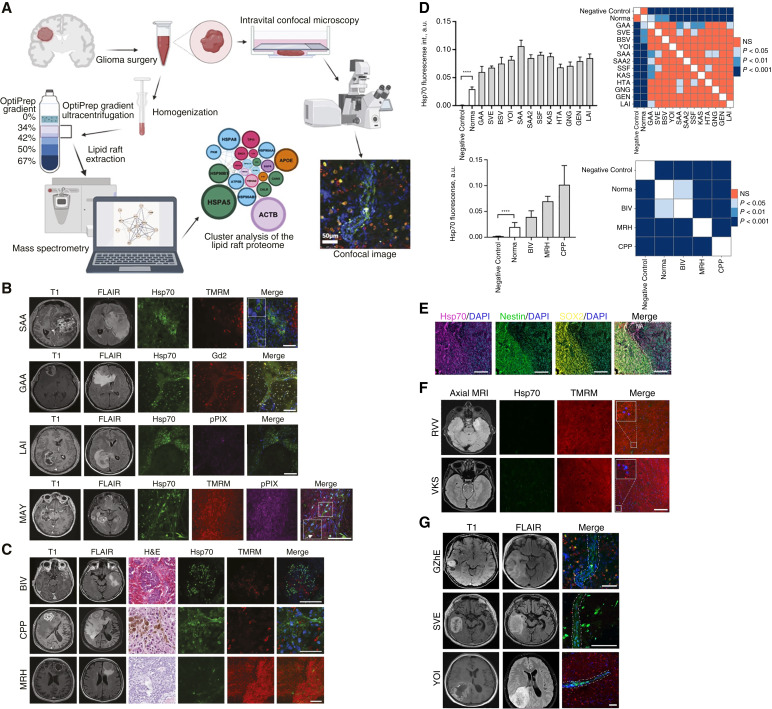
Evaluation of the membrane-bound Hsp70 expression in malignant brain tumors by live-cell confocal microscopy imaging. **A,** Schematic description of the study of brain tumor samples to determine the expression of membrane-associated chaperone in cancer cells, followed by mass spectrometry analysis of isolated lipid rafts. **B,** Representative confocal microscopy images of the brain tumor samples stained for Hsp70 (green), TMRM (tetramethylrodamine methyl ester; red), disialoganglioside GD2 (red), and protoporphyrin IX (pPIX; magenta). Nuclei were stained by DAPI. Scale bars, 100 μm. Respective MR images (postcontrast T1-weighted and FLAIR images) of the patients are presented. **C,** Representative confocal microscopy images of the brain metastasis stained for Hsp70 (green), TMRM (tetramethylrodamine methyl ester; red). Nuclei were stained by DAPI (blue). Scale bars, 100 μm. Respective MR images (postcontrast T1-weighted and FLAIR images) of the patients as well as hematoxylin and eosin staining of tumor samples are presented. **D,** Top row—Fluorescence intensity measurements of mHsp70 expression in live-cell microscopy images of patients with GBM (*n* = 11) with subsequent Benjamini–Yekutieli test for multiple pairwise comparison of protein expression levels. The bar graph shows the median ± 95% CI (Man–Whitney test result—****, *P* < 0.0001). The heat map shows *P* values as the result of a *post hoc* multiple comparison test. Bottom row—Fluorescence intensity measurements of mHsp70 expression in live-cell microscopy images of brain metastasis (*n* = 3) with subsequent Benjamini–Yekutieli test for multiple pairwise comparisons of protein expression levels. The bar graph shows the median ± 95% CI (Man–Whitney test result—****, *P* < 0.0001). The heat map shows *P* values as the result of a *post hoc* multiple comparison test. **E,** Representative multiplex analysis of the immunofluorescence of brain tumor sample stained for Hsp70, SOX2, and nestin. Nuclei were stained by DAPI. NA, necrotic area; VT, vital tissues. Scale bars, 200 μm. **F,** Representative confocal microscopy images of the normal brain tissue samples stained for Hsp70 (green) and TMRM (tetramethylrodamine methyl ester; red). Nuclei were stained by DAPI (blue). Scale bars, 100 μm. Respective MR images of the patients are presented. **G,** Live-confocal microscopy images of mHsp70-positive tumor cells in oncostreams detected in GBM samples. Tumor sections were stained for Hsp70 (green), TMRM (red), and DAPI (blue) for nuclei. Scale bars, 100 μm. For the descriptive characteristics of patients, see Supplementary Table S1.

Expression of the mHsp70 was evaluated by the live-cell inverted confocal microscopy of the intraoperative biopsy material obtained from adult (*n* = 23) and pediatric (*n* = 9) neurooncologic patients as well as brain metastasis (*n* = 3; Fig. 1; Table 1; Supplementary Tables S1–S3). The workflow of the brain tumor sample analysis is depicted in Fig. 1A. Patient-derived tumor samples were obtained from three regions that were designated prior to operation employing MR scanning (as was previously proposed by Hubert and colleagues; ref. 36): (i) tumor center (presented as hypointense on T_1_-weighted images or nonenhancing, which corresponds to the necrotic zone), (ii) the region within the hyperintense FLAIR imaging, and (iii) peritumoral region 5 mm from enhancing margin on the hyperintense FLAIR scanning. Cell viability in the studied tumor material was further monitored by staining the samples with TMRM dye, revealing active mitochondria. Additionally, tumor tissues were costained with fluorophore 5-aminolevulinic acid (5-ALA), which was employed for intraoperative resection of contrast-enhancing tumors and which is known for intracellular accumulation of fluorescent porphyrins in malignant gliomas (54). High heterogeneity in the localization and the level of expression of the studied mHsp70 was revealed within all three studied regions. Figure 1B shows the representative images of biopsy material of patients with glioblastoma, in which mHsp70-positive cancer cells were detected, particularly in the MR contrast-enhancing and peritumoral zones. Additionally, we studied chaperone expression in patients (*n* = 3) with brain metastases (Fig. 1C; Supplementary Table S3) that were also highly positive for mHsp70-positive cells.

Subsequent multiplex immunofluorescence analysis of the tumor sections confirmed that Hsp70 was predominantly expressed in the regions (ii) and (iii) of VTs, colocalizing with the markers of tumor stem cells (i.e., cells expressing stemness markers SOX2, nestin, and Oct4; Fig. 1E). Thus, 67% and 20% double-positive Hsp70+/nestin+ cells were detected in the VT and necrosis, respectively, whereas 35% double-positive Hsp70+SOX2 cells were obtained only a region of VT (Supplementary Figs. S3–S5; Supplementary Table S4). Intriguingly, in the perifocal zone, we detected the mHsp70^+^ oncostreams, which permeated the surrounding normal brain tissue (Fig. 1G). These spindle-like cells formed fascicles of 2 to 4 cells thick and were also positive for protoporphyrin IX (5-aminolevulinic acid metabolite in cancer cells) and disialoganglioside GD2, known to be highly expressed on glioblastoma cells (55). Brain tumor metastasis was highly positive for mHsp70, and upon careful analysis of the images, we were also able to detect oncostreams. For evaluation of mHsp70 expression in the nontumorous brain tissues we employed patient-derived samples obtained intraoperatively from the patients with epilepsy (*n* = 5; Fig. 1F). When live-cell microscopy was performed we detected a light intracellular expression of the chaperone but we did not observe any membrane localization of the mHsp70.

**Figure 2:**
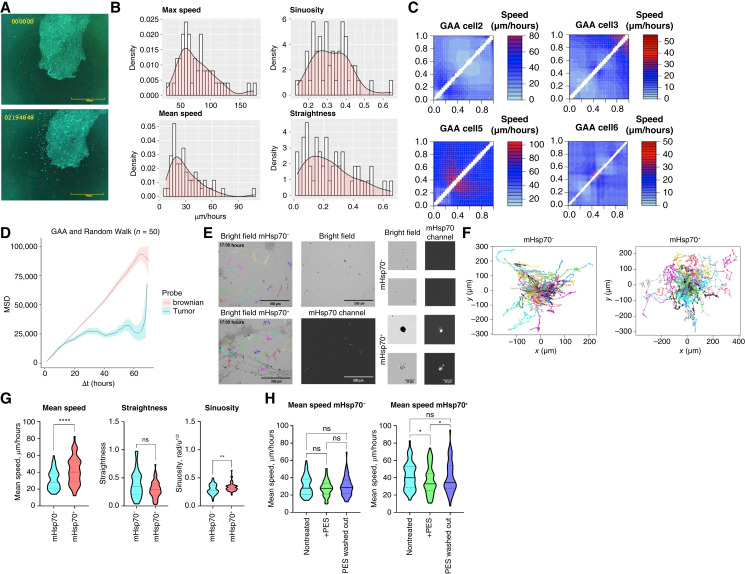
Analysis of the mHsp70-positive tumor cell motility in brain tumors. **A,** Representative confocal microscopy images from 68 hours of time-lapse recording of the tumor sample (patient with GAA) stained for mHsp70 (green). Scale bars, 500 μm. The numbers show the time in the format days:hours:minutes:seconds. **B,** Histograms and density plots of values characterizing tracks of motile cells emerging from a tumor sample: max speed (μm/hour), mean speed (μm/hour), sinuosity, and straightness. **C,** Staggered analysis plots show the changes in cells’ mean speed during 68 hours; four cells with different motility patterns are shown. **D,** MSD plots on tracks (*n*_cells_ = 50) of 68 hours tumor cell motility in comparison with a simulated random walk. **E–H,** Migration characteristics of mHsp70^+^ and patient with mHsp70^−^ IBD explant cell subpopulations. **E,** Microscopy images of explant. From top to bottom: brightfield image with marked tracks of mHsp70^−^ and mHsp70^+^ cells, brightfield image, and corresponding image of mHsp70 fluorescence channel. **F,** Zoomed in samples of microscopy images. **C,** Rose plots of patient with IBD explant cell subpopulations. **G,** Comparison of mean speed, straightness, and sinuosity of subpopulations (*n*_tracks_ ≥ 51). **H,** Comparison of the mean speed of nontreated and treated subpopulations with 20 μmol/L PES (*n*_tracks_ ≥ 59). Data are presented as median ± 95% CI. Significant differences identified by the Wilcoxon test are shown as *, *P* < 0.05; **, *P* < 0.01; ***, *P* < 0.001; ****, *P* < 0.0001; ns, not significant.

Considering the high level of protein expression in the contrast-enhancing region of tumors and in oncostreams, we assumed that the cells expressing it may have high motility and decided to test this hypothesis *ex vivo* on tumor samples (patient GAA, patient IBD) that were stained for mHsp70. Highly motile Hsp70-positive cells with subdiffusive motion were found in this sample (Fig. 2A–C; Supplementary Fig. S6; Supplementary Videos S1–S3) that further supported our hypothesis of mHsp70 involvement in tumor cell motility. Interestingly, in GBM 3D explant sample IBD we detected a cell subpopulation with glial-like morphology positively stained for mHsp70 (Fig. 2E–G). These mHsp70^+^ cells moved with a high mean speed and high sinuosity when compared with the mHsp70^−^ cells (Fig. 2G). Thus, the mean speed for mHsp70^+^ cells constituted 40.21 [34.24; 50.37] μm/hour, whereas the speed for mHsp70^−^ cells was 28.22 [25.31; 35.70] μm/hour (*P* < 0.0001). The sinuosity parameter for chaperone-positive cells constituted 0.32 [0.30; 0.35] rad/u^1/2^, whereas for mHsp70^−^ cells it was 0.28 [0.26; 0.33] rad/u^1/2^ (*P* = 0.0047). The data obtained may indicate that mHsp70 associated with the plasma membrane of cells may participate in the processes of cell invasion. Next, we studied the migration of tumor cells IBD in the presence of Hsp70 inhibitor PES (Fig. 2H). The addition of an inhibitor resulted in a mean speed decrease of the mHsp70^+^ cell subpopulation (*P* = 0.0271; Fig. 2H). The mean speed for mHsp70^+^ following application of PES inhibitor was 34.34 [31.92; 45.19] μm/hour as compared with untreated cells—40.03 [33.50; 50.37] μm/hour. Subsequently, following washing from the inhibitor, we detected the recovery of the mean speed of mHsp70^+^ cells (*P* = 0.0479). The effect of the PES inhibitor on the migratory activity of the mHsp70^−^ cell subpopulation was not observed.

### Membrane-bound Hsp70 is localized in lipid rafts of the brain tumor cells in an HSP cluster

**Figure 3:**
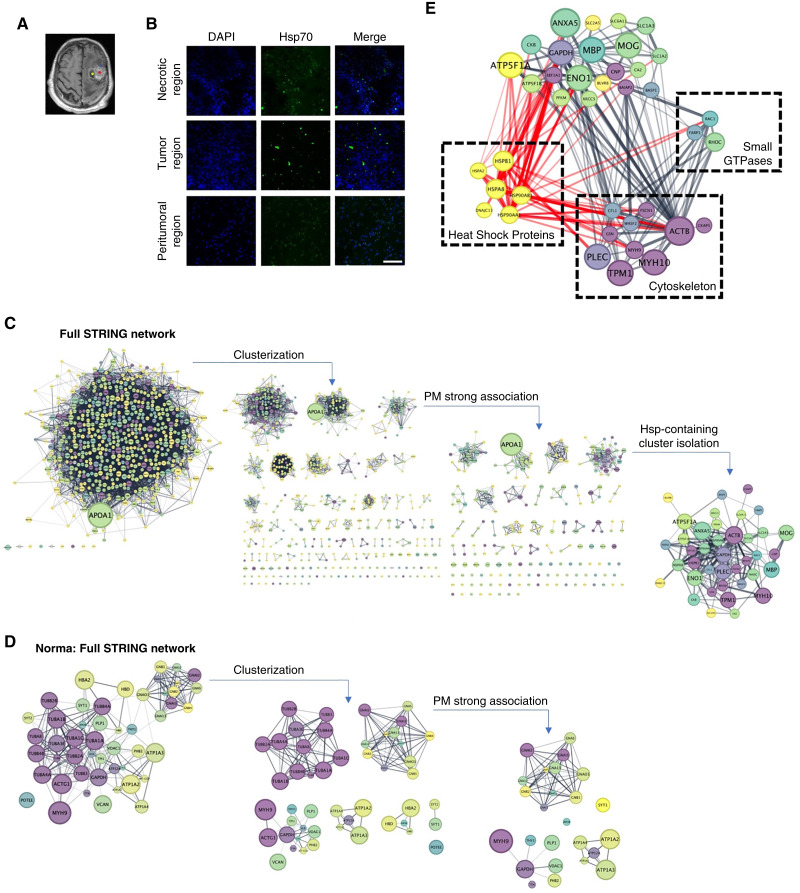
Mass spectrometry analysis of the tumor-derived lipid rafts. **A,** Designation of the brain tumor regions on postcontrast T1-weighted image for obtaining tumor samples from necrotic, tumor, and peritumoral regions. **B,** Representative confocal microscopy images of the brain tumor samples obtained from three regions and stained for mHsp70 (green) and DAPI (blue) for nuclei. Scale bars, 100 μm. **C–E,** Analysis of the mass spectrometry data from isolated lipid rafts. Protein functional groups identified using the STRING database in the proteome of lipid rafts from three tumor zones (Supplementary Fig. S7). **C,** A subnetwork of highly membrane-associated proteins isolated from the tumor-derived lipid raft and **D,** normal tissue-derived lipid raft STRING PPI proteome network, separated into clusters using the Markov cluster algorithm method. Color mapping (light to dark) was done to indicate the nodes belonging to the cytoskeletal compartment, the size of the node corresponding to the protein abundance index obtained from mass spectrometry data. **E,** The cluster containing highly plasma membrane-associated proteins contains simultaneously heat shock proteins, small GTPases, and cytoskeletal proteins.

To further confirm the presence of mHsp70 in the plasma membrane of tumor cells we have extracted the lipid rafts from patient-derived samples and performed a mass-spectometry analysis (Fig. 3A and B; Supplementary Fig. S7). A total of 638 proteins have been identified. All samples contain flotillin, a marker of lipid rafts. The data clearly demonstrated the expression of mHsp70 in all studied samples. Interestingly, we also revealed a cluster containing heat shock proteins (Hsp27, Hsp40, Hsp70, and Hsp90), small GTPases with GEF (Rac1, RhoC, and FARP1), and several cytoskeletal proteins, including actin, myosin-9 cytoskeletal protein, gelsolin, fascin, cofilin, and plectin (Fig. 3C–E). In the proteome of lipid rafts from normal tissue, MS analysis identified 50 proteins (Supplementary Table S5), among which neither heat shock proteins nor small GTPases were detected (Fig. 3D).

### mHsp70 is associated with a high migratory potential of brain tumor cells

**Figure 4:**
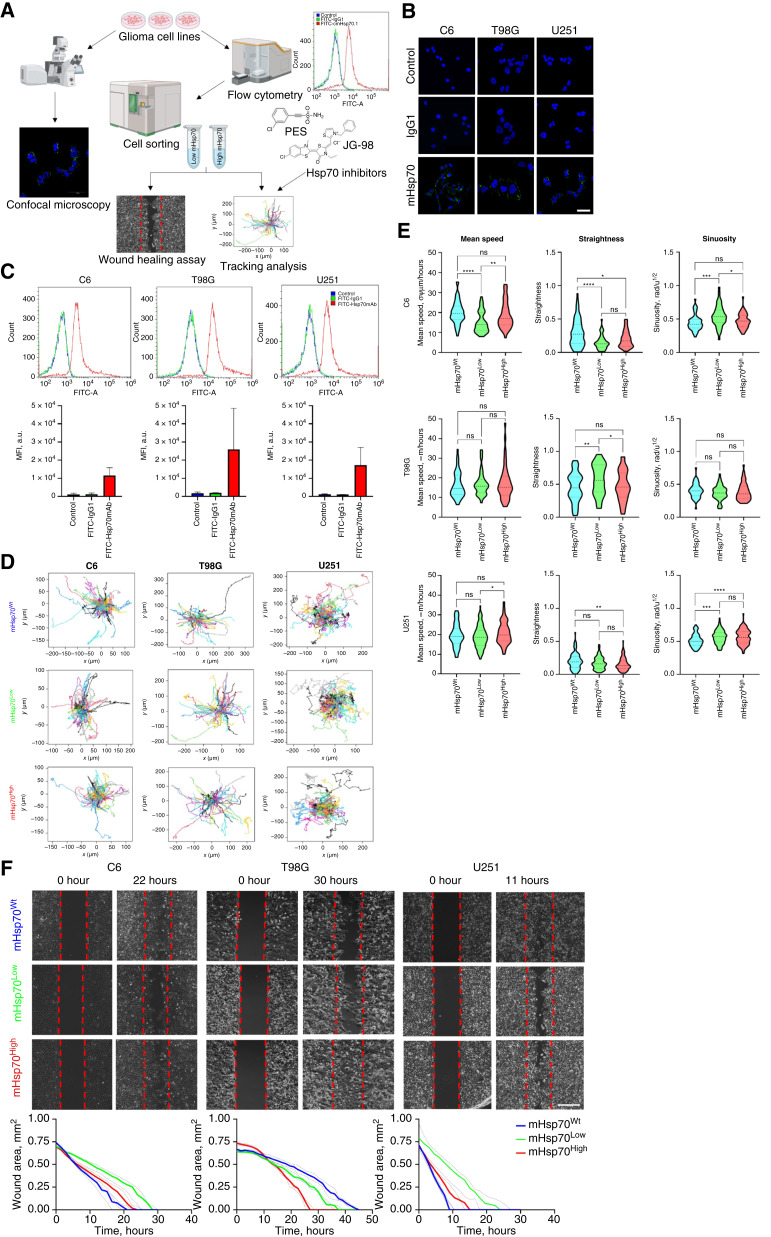
Motility analysis of sorted C6, T98G, and U251 cell lines by the level of mHsp70 expression. **A,** Representative scheme of mHsp70 expression analysis and FACS for subsequent determination of cell migration characteristics. **B,** Confocal microscopy images of C6, T98G, and U251. DAPI was applied for nucleus staining (blue). FITC-IgG1 and FITC-anti-Hsp70 antibody was used for detecting mHsp70 on the plasma membrane (green). Scale bars, 50 μm. **C,** Mean fluorescence intensity of FITC-anti-Hsp70 antibody in arbitrary units measured by flow cytometry (*n* = 4). **D,** Example of tracks rose plots in motility analysis. **E,** C6, T98G, and U251 cell tracks violin plots. **F,** Phase contrast live images of wound healing assay of subpopulations with different mHsp70 expression and plots of wound area vs. healing time. Scale bars for images, 400 μm.

To evaluate the involvement of mHsp70 in the tumor cell migration we have sorted brain tumor cells C6, T98G, and U251 known to be positive for the membrane-bound chaperone (based on the confocal microscopy and FACS analysis studies; Fig. 4) into two subpopulations with high (mHsp70^High^) and low (mHsp70^Low^) expression of the protein (Fig. 4A). Thus, the MFI for mHsp70^High^ cells were 10-fold higher than mHsp70^Low^ cells (Fig. 4B and C). We assessed the motility characteristics (i.e., mean speed, straightness, and sinuosity) of cellular subpopulations employing long-term live-cell imaging with the CellVoyager CQ1 Benchtop High-content Analysis System (Yokogawa, Japan; Fig. 4D and E). For human U251 glioblastoma and rat C6 glioma cells, it was shown that the subpopulation of mHsp70^High^ cells moved significantly faster than the mHsp70^Low^ cells (*P* = 0.0139 and *P* = 0.0054, respectively; Fig. 4D and E). Thus, the mean speed median ± (95% CI) for mHsp70^High^ U251 cells constituted 19.8 [18.7; 21.7] μm/hour, whereas the speed for mHsp70^Low^ and mHsp70^Wt^ cells constituted 18.6 [17.1; 19.6] and 19.0 [18.0; 20.3] μm/hour, respectively. At the same time, subpopulations differed in the movement pattern: the sinuosity of the mHsp70^High^ and mHsp70^Low^ U251 and C6 cells tracks increased compared with the mHsp70^Wt^, whereas the straightness decreased. Statistical analysis of the mean speed and sinuosity of the T98G cell subpopulations did not reveal significant differences in these parameters. Subsequently, to assess the contribution of mHsp70 to cell migration, a wound healing assay was performed for these two subpopulations of three cell lines. As demonstrated in Fig. 4F application of mHsp70^High^ cells led to complete wound healing in a shorter period of time as compared with mHsp70^Low^ cells.

**Figure 5:**
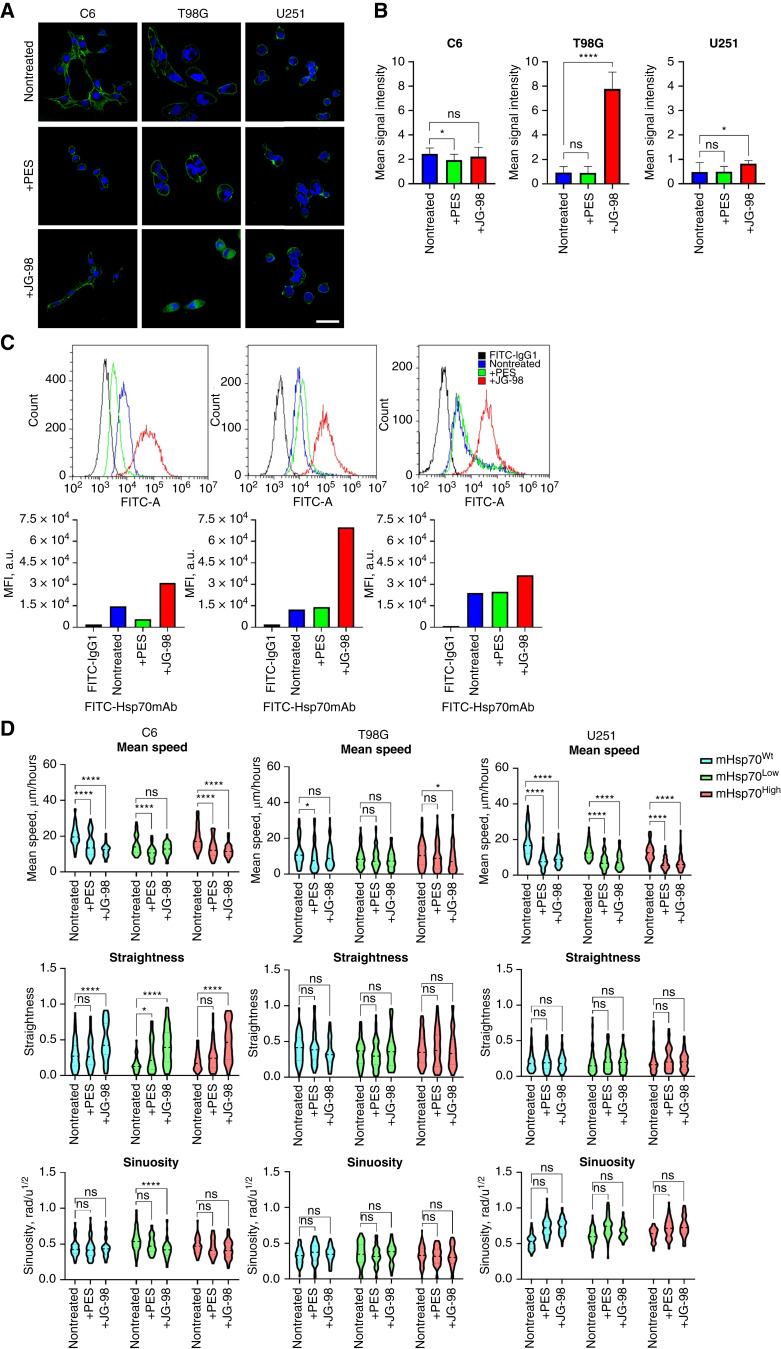
Application of Hsp70 inhibitors decreases the brain tumor cell migratory activity. **A–C,** mHsp70 expression in nontreated C6, T98G, U251 cell lines and cells incubated with 1 μmol/L PES or 50 nmol/L JG98 inhibitors. **A,** Confocal microscopy images of C6, T98G, U251. DAPI was applied for nucleus staining (blue). FITC-anti-Hsp70 antibody was used for detecting mHsp70 on the plasma membrane (green). Scale bars, 50 μm. **B,** Mean signal intensity of FITC-anti-Hsp70 antibody in nontreated C6, T98G, U251 cell lines and cells incubated with 1 μmol/L PES or 50 nmol/L JG98 inhibitors observed by confocal microscopy (*n*_images_ ≥ 20). **C,** Mean fluorescence intensity of FITC-anti-Hsp70 antibody in arbitrary units measured by flow cytometry. **D,** Comparison of mean speed, straightness, and sinuosity of sorted nontreated and treated C6, T98G, and U251 cells with 1 μmol/L PES or 50 nmol/L JG98 (*n*_tracks_ ≥ 46). Data are presented as median ± 95% CI. Significant differences identified by the Wilcoxon test are shown as *, *P* < 0.05; **, *P* < 0.01; ***, *P* < 0.001; ****, *P* < 0.0001; ns, not significant.

To evaluate the contribution of either substrate- or nucleotide-binding domain of mHsp70 in the tumor cells migration, we employed specific inhibitors directed to each of the domains—PES and JG98 at the concentrations nontoxic (Supplementary Fig. S8) for cells (56, 57). Addition of a PES inhibitor did not increase the mHsp70 level (as shown by confocal microscopy and FACS; Fig. 5A–C). However, when JG98 was applied we observed at least a twofold increase in the membrane-bound chaperone. PES and JG98 application also contributed to a change in such migration characteristics as straightness and sinuosity. When inhibitors were applied, we observed a significant reduction in the mean speed for all studied cell lines (i.e., C6, T98G, and U251). Intriguingly, the effect of inhibitors was more pronounced for mHsp70^High^ subpopulations, which is consistent with our hypothesis (Fig. 5D).

**Figure 6:**
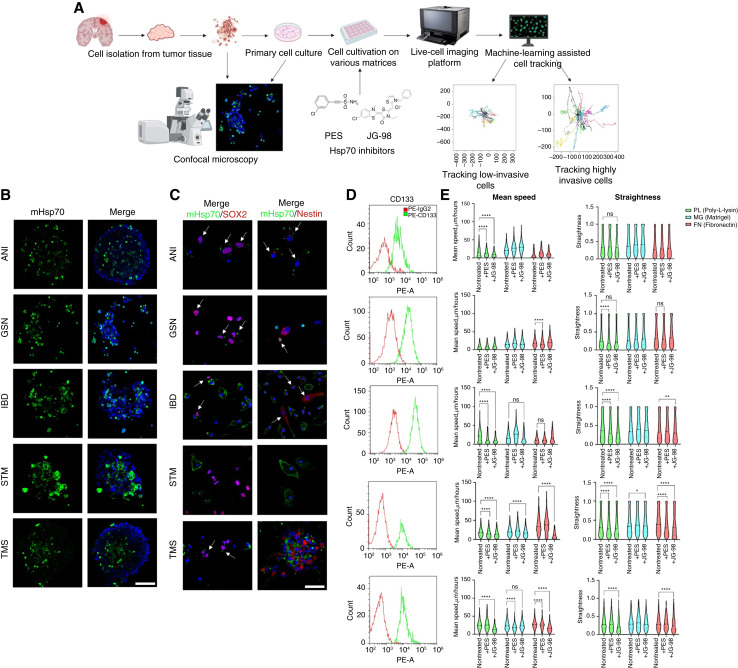
Inhibition of mHsp70 results in decreased motility of patient-derived brain tumor cells. **A,** The process of isolating the primary human GBM cell line from postoperative material, obtaining confocal images, shooting on a live-cell imaging platform with the addition of inhibitors, and subsequent training and implementation of an AI model for tracking highly invasive cells. **B** and **C,** Representative confocal microscopy images of the patient-derived tumor cell lines stained for mHsp70 (green), SOX2 (magenta), and nestin (magenta). Nuclei were stained by DAPI (blue). Scale bars, 100 μm. **D,** CD133 expression in patient-derived tumor cells stained by PE-anti-CD133 antibody (as measured by flow cytometry). **E,** Comparison of mean speed and track straightness on various matrices (PL, MG, and FN) of patient-derived high-speed brain tumor cells treated with 1-μmol/L PES or 50-nmol/L JG98 (*n*_tracks_ ≥ 294). Nontreated cells were used as a control. Data are presented as median ± 95% CI. Significant differences identified by the Wilcoxon test are shown as *, *P* < 0.05; **, *P* < 0.01; ***, *P* < 0.001; ****, *P* < 0.0001; ns, not significant.

Additionally, we studied the involvement of mHsp70 in cell migration on the glioblastoma cells obtained from the neurooncological patients with a newly diagnosed glioblastoma (*n* = 5; Fig. 6A–E). Microscopy studies for the detection of mHsp70 revealed the presence of this protein on the cell surface in all samples (Fig. 6B). Moreover, costaining of the tumor cells for SOX2 and nestin demonstrated that mHsp70 was also abundantly expressed (as well as CD133) on the tumor stem cells positive for these markers (Fig. 6C and D; Supplementary Figs. S9 and S10). Taking into account the fact that it is cancer cells with high migratory activity that have increased expression of mHsp70, we applied machine learning and divided the primary GBM cells into two subpopulations [cells with higher migratory activity (high-speed) and cells with lower migratory activity (low-speed)] and further assessed the effect of the inhibitors on them (Fig. 6E; Supplementary Fig. S11; Supplementary Videos S4–S6).

To further reconstruct tumor microenvironment cells were cultured on various matrices (i.e., poly-L-lysine, Matrigel, and fibronectin). As expected, we did not reveal a significant effect of HSP70 inhibitors on low-speed cells that also exhibit negative Hsp70 staining. Notably, high-speed tumor cell subpopulations showed various response rates to the applied PES or JG98 inhibitors. Thus, for patients with STM, we observed a significant reduction of mean speed from 24.65 [24.30; 25.08] to 12.67 [11.93; 13.39] (PL) μm/hour, 23.29 [22.83; 23.83] to 23.00 [22.48; 23.46] (MG) μm/hour, and 27.00 [26.48; 27.44] to 17.99 [17.29; 18.63] (FN) μm/hour particularly when JG98 inhibitor was employed (Fig. 6E). Other patient cell lines (ANI, GSN, IBD, and TMS) showed varying sensitivity to either PES inhibitor or JG98 inhibitor (in terms of mean speed and straightness) depending on the used matrix for culture.

### Inhibition of Hsp70 resulted in increased overall survival of glioma-bearing animals

**Figure 7:**
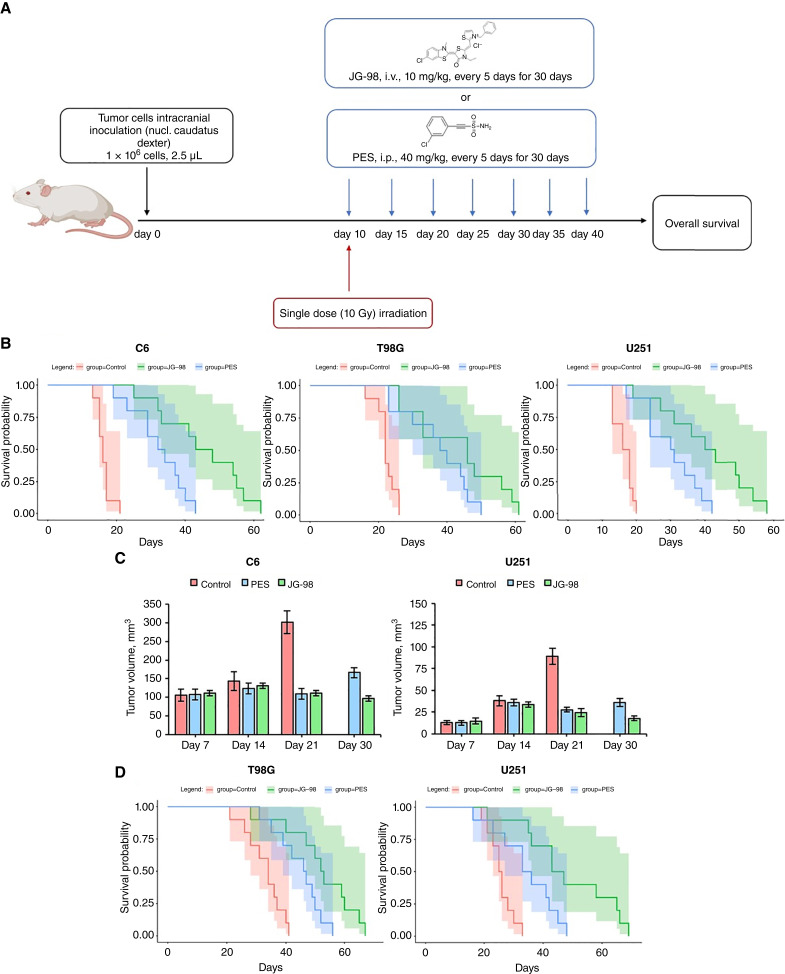
Therapeutic efficacy of the HSP70 inhibitors PES and JG98 in orthotopic brain tumor models. The survival curves of the glioma-bearing animals were analyzed employing the Kaplan–Meier method. **A,** Schematic representation of the preclinical study protocol. **B,** Kaplan–Meier curves of animals treated with PBS, PES, or JG98. **C,** Volumetric studies of the C6 rat glioma and U251 human glioblastoma. Tumor volume (mm^3^) was determined by employing T_1_-weighted and T_2_-weighted MRI scans in tumor-bearing animals (*n* = 6 per group). Data are presented as mean ± SD. **D,** Kaplan–Meier curves of animals treated with PBS, PES, or JG98 in combination with a single-dose (10 Gy) irradiation.

Employing intracranial glioma animal models, we examined whether treatment with chaperone inhibitors PES or JG98 could induce the therapeutic effect (Fig. 7A). Administration of PES intraperitoneally every 5 days (for 30 days, 40 mg/kg) starting on day 10 resulted in the increased animal OS for C6 rat glioma in Wistar rats and human T98 and U251 gliomas in SCID mice as compared with control untreated group, constituting 32.4 ± 7.6, 37.5 ± 9.5, 30.2 ± 7.9 days (*P* < 0.001), respectively [in control groups—16.2 ± 2.1 (C6), 22.3 ± 2.9 (T98), 16.4 ± 2.6 (U251) days; Fig. 7B]. When JG98 was applied intravenously 10 mg/kg (every 5 days for 30 days) starting on day 10 after tumor cells inoculation, we observed further prolongation of animal survival as compared with the PES-treated groups—45.0 ± 12.3 (C6; *P* = 0.015), 43.4 ± 13.2 (T98), and 40.7 ± 12.5 (U251; *P* = 0.04) days (Fig. 7B). Additionally, we evaluated the tumor growth employing MRI tumor volumetrics (mm^3^) in animals bearing C6 or U251 glioma (Fig. 7C). Thus, on 21st day following C6 glioma implantation in the control group, the tumor volume constituted 301 ± 31 mm^3^, whereas in PES- and JG98-treated groups, the tumor volume was significantly reduced—109.2 ± 8.6 and 110.8 ± 10.9 mm^3^, respectively. On the 30th day decrease in the tumor volume was further detected in the JG98-treated group—96.1 ± 8.9 mm^3^, as compared with the PES-treated animals (166.4 ± 14.1 mm^3^). The same effect was observed in U251-bearing mice. On the 21st day in the control group, the tumor volume was 88.9 ± 9.3 mm^3^, whereas in the PES- and JG98-treated groups, the tumor volume was significantly decreased—27.9 ± 2.5 and 24.6 ± 4.5 mm^3^. Next, we assessed whether the combination of anti-Hsp70 therapy with a single dose (10 Gy) irradiation might further increase the animal survival for T98 and U251 models (Fig. 7D). We detected a prolongation of OS when inhibitors were combined with tumor irradiation, constituting for PES—44.7 ± 7.9 (T98), 34.4 ± 10.2 (U251) days, and for JG98—52.1 ± 11.8 (T98), 48.3 ± 15.8 (U251) days. However, subsequent intergroup statistical analysis did not reveal the benefit of the combined treatment over the monotherapeutic regimens. Notably, when combined therapies—irradiation + PES and irradiation + JG98—were compared with each other we observed a statistical significance of *P* = 0.03.

## Discussion

Malignant brain tumors, particularly glioblastomas, have a high degree of invasiveness, which is attributed to the high recurrence rate of tumors and ineffective current treatment modalities that in turn results in the poor median overall survival of patients of only 12 to 18 months (58–60). Recent studies indicate that chaperones might be involved in tumor cell invasion and metastasis, which forces one to pay closer attention to the participation of these proteins in the described invasion processes. Live-cell confocal microscopy of the studied brain tumor samples from neurooncologic patients demonstrated the high expression of mHsp70 on the surface of cancer cells, particularly from the areas of perifocal zone and live tissues (i.e., contrast-enhancing zone as visualized by MRI) but not in the necrotic tumor regions (Fig. 1). Indeed, as reported previously, only live cells (that can be exposed to nonlethal stress conditions) but not necrotic cells do upregulate mHsp70 expression (61, 62). Notably, subsequent multiplex histologic studies demonstrated not only the predominant presence of Hsp70 in live glioma tissues but also its colocalization in tumor stem cells (as described by CD133, SOX2, and nestin stemness markers coexpression; Figs. 1E and 6). One could assume that not only HSP70 but also other representatives of the HSP family might play a role in the maintaining of the glioma cells’ stemness. Thus, it was demonstrated by Park and colleagues (63) that interplay between TRAP1 mitochondrial chaperone and SIRT3 (mitochondria deacetylase sirtuin-3) increased adaptation to stress and maintained the stemness of the glioblastoma cells.

Taking into account the fact that chaperones are present in the plasma membrane as part of lipid rafts (8, 64), for a more detailed analysis of the expression of mHsp70 and other possible representatives of the HSPs family, we isolated rafts from tumor tissue samples and conducted a mass spectrometry study (Fig. 3). As expected, we detected HSP70 in all analyzed samples. However, in addition to this protein, in all samples, we also identified representatives of other families—Hsc70, Hsp90, and Hsp105. At the same time, chaperones were not identified in lipid rafts from patients without cancer (epilepsy), which we conventionally considered to be a healthy norm. It can be assumed that to ensure the processes of cell motility and possibly other cell functions (e.g., association with the extracellular matrix and transport of molecules across the membrane), the presence of the entire assembly of representatives of heat shock proteins is necessary for the full functioning of the chaperone machinery on the surface of the plasma membrane. A strong association of Rac1 GTPase with solid tumors is known from the literature (65). We hypothesize that membrane-bound heat shock proteins can stabilize lipid rafts, thereby maintaining enhanced signaling from raft-associated Rho family small GTPases, which allows cells to realize a highly invasive phenotype. This hypothesis is supported by the discovery of the small GTPases Rac1 and RhoC, as well as the cytoskeletal protein myosin-9, in lipid rafts isolated from GBM.

Intriguingly, upon closer examination of the obtained live-confocal microscopy images (particularly in the tumor perifocal zone), we were able to discover multicellular fascicles of spindle-like cells highly positive for mHsp70 (Fig. 1G). These longitudinal strings are only two or three parallel cells thick and similar to one recently described by Comba and colleagues (66) who demonstrated that glioma tumors do contain dynamic aligned cell flows (termed oncostreams) that are correlated to glioblastoma aggressiveness (as shown in genetically engineered mice GBM models). These mHsp70-positive oncostreams were also observed in patients with brain metastasis (*n* = 3), which themselves expressed quite high levels of mHsp70 as compared with primary GBM (Fig. 1C).

Interestingly, when a tumor sample from a patient with GAA was cultured for 3 days, motile cells emerged from the sample only after 1 day of cultivation (Fig. 2; Supplementary Video S3). Apparently, the highly invasive cells needed the first 24 hours to adapt to the culture conditions. As can be seen from the staggered analysis plots, the Hsp70-positive cells emerging from the tumor sample had different patterns of movement; some could increase their speed at the end of the third day of cultivation. The anomalous diffusion of tumor cells may be an important feature for characterizing highly invasive cells (67). We discovered in the experiment the subdiffusion nature of the motility of tumor cells in culture. Subdiffusion movement of cells emerging from the tumor sample of a patient with GAA can be explained by the fact that, under culture conditions, a gradient of extracellular matrix components is formed with a maximum concentration closer to the tumor piece.

Taking into account the facts of increased expression of the membrane-bound chaperone mHsp70 in the perifocal region (as well as on cells of brain metastases), on the surface of cells as part of oncostreams, we decided to study in more detail the contribution of the protein to the motility of cancer cells. Using various cancer cell lines (C6, T98, and U251) sorted by the level of mHsp70 expression into high (mHsp70^+^) and low (mHsp70^−^) expressing, we demonstrated that the degree of protein expression also determines higher cell motility (Fig. 4). The inhibitory assay further confirmed that both domains of the membrane-exposed chaperone contribute equally to cell motility. The next step was to test the effect of the inhibitors on primary cultures from patients (which were also cultured on different matrices to reproduce the conditions of exposure to the tumor matrix; Fig. 6). It turned out that the highest therapeutic effect was observed when exposed to the JG98 inhibitor, whereas different sensitivity of cells from patients to its action was noted. Presumably, it is the disruption of the functioning of the N-terminal domain of the chaperone that determines the protein’s contribution to cell migration.

One of the plausible mechanisms explaining the association of mHsp70 with an increased cancer cell metastasis and invasion could be the involvement of chaperones in mediating the function of other molecules responsible for extracellular matrix (ECM) remodeling (modifying such enzymes as matrix metalloproteinases, plasminogen activator, lysyl oxidase proteins, heparanase, and cathepsins; ref. 68). Indeed, as was shown previously, extracellular Hsp70 and Hsp90α assist the function of the matrix metalloproteinase 2, plasminogen activator, lysyl oxidase 2-like protein, and fibronectin, whereas the inhibition of chaperones resulted in the decreased cancer cell migration (69–72). Presumably, other extracellular members of the HSP family [i.e., Hop (Hsp70/Hsp90 organizing protein), Hsp40, p23, and Hsp27] are also involved in the modulation of the ECM enzyme activity (69, 73). Intriguingly, the mass spectrometry analysis of the isolated lipid rafts with subsequent interactome analysis revealed a strong association of HSP cluster with small GTPases (Rac1) that are known to be involved in the cell migration and invasion (Fig. 3). Presumably, the chaperone machinery on the surface of the plasma membrane ensures the maintenance of the structure and work of other enzymes, protein kinases, etc., which in turn determine cell migration. The task of subsequent studies will be to study the direct relationship of anchored chaperones in lipid rafts with the components of the ECM, their role in maintaining the architecture of the matrix and the migration of cancer cells in it as well as to reveal the role of HSP cluster in the maintenance of other proteins.

In the current study, we showed that mHsp70 might contribute to the invasive potential of the GBM cells, which makes it possible to use this protein as a target for the development of antitumor treatment. Indeed, recent preclinical studies clearly demonstrated the theranostic potential of mHsp70 in the management of tumors when various chaperone-targeted molecules (e.g., monoclonal antibodies, anticalins, and peptides) were applied (10, 74–78). For our *in vivo* study employing orthotopic glioblastoma models, we applied either PES or JG98 inhibitor treatment (Fig. 7). Subsequent analysis demonstrated the efficacy of both monotherapeutic approaches with a pronounced predominance of JG98 inhibitor effectiveness over PES (*P* < 0.001). Presumably, the therapeutic effect of both inhibitors apart from the reduction in tumor cell invasion and migration could be also attributed to the reduction of cell viability. The obtained data are in accordance with previously reported data by Wong and colleagues (79) who showed the efficacy of JG98 in the xenograft human colorectal cancer model by reducing tumorigenicity and metastatic activity.

As was shown in the study by Xie and colleagues (80) Hsp70-targeting by self-assembled gold nanoparticles containing doxorubicin (D-A-DA/TPP) in combination with the PD1 checkpoint blockade increased the chemotherapeutic drug enrichment in glioma that further potentiated the immunotherapy. Thus, targeting mHsp70 can be included as an adjuvant method in existing standard approaches to glioblastoma therapy, although additional clinical studies are required to test this hypothesis.

In our study, we evaluated the feasibility of the combination of anti-Hsp70 therapy with radiotherapy. Of note, several factors including γ irradiation and chemotherapeutic agents applied to the tumor cells result in an upregulation of cytosolic and membrane-bound HSPs in tumor cells (8). An increase in protein expression on the surface of the plasma membrane leads to an increase in target density for the targeted drugs applied and, as a result, enhances the sensitivity of cancer cells to them. Thus, in a previous study on a model of intracranial glioma in rats, it was shown that a single dose (10 Gy) irradiation of the tumor led to a substantial increase in Hsp70 expression on the membrane and a more than sevenfold increase in the accumulation of anti-mHsp70 targeted nanoparticles in the tumor tissue (74). In a recent study, we showed that irradiation of cancer cells creates a “*therapeutic window*” when, after irradiation, an increase in mHsp70 expression is observed for approximately 7 days, which also increases the effectiveness of anti-HSP70 therapy (81). This fact provides a scientific basis for the combination of anti-HSPs-targeted therapies with conventional radiotherapy in the treatment of GBM. Indeed, we observed an increased animals’ OS when either PES or JG98 inhibitor was applied in combination with single dose (10 Gy) radiotherapy (Fig. 7). However, the statistical analysis did not show any benefit of the combined treatment over the monotherapeutic modality. Notably, when both combined regimens were compared (PES + radiation vs. JG98 + radiation), we observed a statistical difference in favor of the JG98 inhibitor (*P* = 0.03). Presumably, to further enhance the therapeutic potency of the agent one should increase the administered dose and period of treatment.

In conclusion, the membrane-bound mHsp70 protein in cancer cells is associated with the formation of a subpopulation of the cell pool, which has increased invasive and migratory activity and may be reflected in the appearance of oncostreams in glioblastoma tissue and metastases to the brain. It is worth noting that the protein itself is present in lipid rafts in close connection with the chaperone cluster (including other members of the HSP family), which indicates the need for further studies of the role of the protein in cellular processes of invasion and migration in a comprehensive proteomic analysis of other HSPs (as well as their interactome networks). Perhaps the formed functional chaperone networks on the surface of the cell membrane determine the behavior of the cancer cell. The use of protein inhibitors, especially those targeting its nucleotide-binding domain (JG98), has shown its therapeutic effectiveness at the cellular level and in models of intracranial tumors, opening up the possibility of using drugs as possible adjuvants in the treatment of patients with cancer. Subsequent research in the field of pharmacology will probably have to focus on the creation of membrane-selective forms of inhibitors, which will avoid possible side effects from antichaperone therapy.

## Supplementary Material

Supplementary Table S1 Characteristics of adult patients with high-grade gliomas.

Supplementary Table S2 Characteristics of pediatric patients with high-grade gliomas.

Supplementary Table S3 Characteristics of patients with brain metastases.

Supplementary Table S4 Grade content and co-localization of biomarkers (Hsp70, Nestin, SOX2) on histological preparations of human GBM.

Supplementary Table S5 List of proteins detected by mass spectrometric analysis of lipid rafts isolated from normal brain tissue (obtained from a patient with epilepsy).

Supplementary Figure S1 Gating of mHsp70High cells (field R2) and mHsp70Low cells (field R3) by FACS.

Supplementary Figure S2 CD133 expression in C6, T98G and U251 cell lines measured by flow cytometry.

Supplementary Figure S3 Preparing a tumor sample for confocal microscopy.

Supplementary Figure S4 The sequence of constructing a phenotyping map for histological sections of human GBM.

Supplementary Figure S5 Evaluation of biomarkers co-localization.

Supplementary Figure S6 Motility of Hsp70-positive cells in a tumor sample.

Supplementary Figure S7 Analysis of the mass-spectrometry data from isolated lipid rafts.

Supplementary Figure S8 Effect of Hsp70 inhibitors PES and JG-98 on cell viability analyzed using the MTT assay.

Supplementary Figure S9 Confocal microscopy images of primary glioblastoma cells stained for mHsp70 and SOX2.

Supplementary Figure S10 Confocal microscopy images of primary glioblastoma cells from patients stained for mHsp70 and Nestin.

Supplementary Figure S11 Comparison of mean speed and track straightness on various matrices (PL, MG, FN) of patient-derived (non-sorted into high- and low-speed subpopulations) brain tumor cells treated with 1 µM PES or 50 nM JG-98.

Video S1 Motility of Hsp70-positive cells in a GBM tumor sample detected using laser scanning confocal microscopy during observation for 1 h.

Video S2 Motility of Hsp70-positive cells in a GBM tumor sample detected using laser scanning confocal microscopy during observation for 1 h, zoomed field of view from the center of the Video S1 frame.

Video S3 Motility of Hsp70-positive cells emerging from a piece of tumor during cultivation for three days.

Video S4 Migration of cells out of spheroids. TMS cell line, a well without the addition of inhibitors on a Poly-L-lysine matrix.

Video S5 Automatic cell tracking before the AI model is applied. TMS cell line, a well without the addition of inhibitors on a Poly-L-lysine matrix.

Video S6 Cells of the TMS cell line in the well with the addition of a PES inhibitor on the matrigel matrix.
