# Supplementary material for: Membrane-bound Heat Shock Protein mHsp70 Is Required for Migration and Invasion of Brain Tumors
Source: Cancer Res Commun. 2024 Aug 12;4(8):2025–44. doi: 10.1158/2767-9764.CRC-24-0094 (PMC11317918; doi:10.1158/2767-9764.CRC-24-0094)
Supplement: Supplementary Table S1 — Characteristics of adult patients with high-grade gliomas. [file crc-24-0094_supplementary_table_s1_suppst1.docx]

| **Patient code** | **Age (years)** | **Sex** | **KPS before surgery** | **Dexamethasone before surgery (mg)** | **Tumor site (lobe)** | **Hemisphere** | **Histology** | **index Ki-67 (%)** | **MGMT** | **ATRX** | **EGFR** | **FGS** |
| --- | --- | --- | --- | --- | --- | --- | --- | --- | --- | --- | --- | --- |
| **ANI** | 57 | male | 70 | 12 | frontal | left | GB  IDH-wt | 20 | ++ | ++ | focal | no |
| **GSN** | 44 | male | 60 | 12 | frontal | right | GB  IDH-wt | 35 | ++ | ++ | focal | no |
| **GAA** | 60 | male | 90 | 12 | frontal | right | GB  IDH-wt | 15 | +++ | + | focal | no |
| **LAI** | 47 | male | 80 | 16 | occipital | right | GB  IDH-wt | 20 | ++ | ++ | focal | no |
| **YOI** | 45 | female | 80 | 12 | occipito-temporal | right | GB  IDH-wt | 30 | ++ | + | focal | yes |
| **SVE** | 76 | male | 70 | 8 | temporal | right | GB  IDH-wt | 25 | + | ++ | diffuse | yes |
| **BSV** | 60 | male | 70 | 16 | temporal | left | GB  IDH-wt | 70 | + | +++ | diffuse | no |
| **SFF** | 63 | male | 70 | 12 | parietal | right | GB  IDH-wt | 20 | + | + | focal | no |
| **GNG** | 61 | female | 70 | 12 | frontal | right | GB  IDH-wt | 15 | ++ | ++ | focal | no |
| **GEN** | 73 | male | 60 | 12 | temporal | right | GB  IDH-wt | 20 | ++ | ++ | focal | no |
| **MAY** | 59 | male | 70 | 16 | temporal | right | GB  IDH-wt | 20 | ++ | + | focal | yes |

Notes: KPS - Karnofsky Performance Scale; FGS - Fluorescence-Guided Surgery with 5-aminolevulinic acid; GB – glioblastoma; wt – wild type;

MGMT: + positive nuclear staining in <10% of tumor cells, ++ positive nuclear staining in 10-50% of tumor cells, +++ positive nuclear staining in >50% of tumor cells; ATRX: + positive nuclear staining in single tumor cells, ++ positive nuclear staining < 95% of tumor cells, +++ positive nuclear staining in > 95% of tumor cells; EGFR: focal - positive cytoplasmic staining in <50% of tumor cells, diffuse - positive cytoplasmic staining in ≥50% of tumor cells.

**Supplementary Table S1.** Characteristics of adult patients with high-grade gliomas.
