# Supplementary material for: Membrane-bound Heat Shock Protein mHsp70 Is Required for Migration and Invasion of Brain Tumors
Source: Cancer Res Commun. 2024 Aug 12;4(8):2025–44. doi: 10.1158/2767-9764.CRC-24-0094 (PMC11317918; doi:10.1158/2767-9764.CRC-24-0094)
Supplement: Supplementary Table S2 — Characteristics of pediatric patients with high-grade gliomas. [file crc-24-0094_supplementary_table_s2_suppst2.docx]

| **Patient code** | **Age (years)** | **Sex** | **Dexamethasone before surgery (0 – no; 1 - yes)** | **Tumor site** | **Hemisphere** | **Histology** | **MIB-1 index (%)** | **FGS** |
| --- | --- | --- | --- | --- | --- | --- | --- | --- |
| **SAA** | 14 | male | 1 | temporal | left | pHGG | 9 | no |
| **IBD** | 11 | male | 1 | thalamus | left | pHGG | 20 | no |
| **TMS** | 3 | female | 1 | temporal | left | pHGG | 70 | yes |

Notes: FGS - Fluorescence-Guided Surgery with 5-aminolevulinic acid; pHGG - pediatric high-grade glioma.

**Supplementary Table S2.** Characteristics of pediatric patients with high-grade gliomas.
