# Supplementary material for: Membrane-bound Heat Shock Protein mHsp70 Is Required for Migration and Invasion of Brain Tumors
Source: Cancer Res Commun. 2024 Aug 12;4(8):2025–44. doi: 10.1158/2767-9764.CRC-24-0094 (PMC11317918; doi:10.1158/2767-9764.CRC-24-0094)
Supplement: Supplementary Table S3 — Characteristics of patients with brain metastases. [file crc-24-0094_supplementary_table_s3_suppst3.docx]

| **Patient code** | **Age (years)** | **Sex** | **KPS before surgery** | **Dexamethasone before surgery (mg)** | **Tumor site (lobe)** | **Hemisphere** | **Histology** | **FGS** |
| --- | --- | --- | --- | --- | --- | --- | --- | --- |
| **CPP** | 75 | male | 60 | 16 | frontal | right | mts of melanoma (BRAF negative) | no |
| **BIV** | 59 | male | 70 | 12 | temporal | left | mts of small cell lung carcinoma | no |
| **MRH** | 62 | male | 70 | 12 | frontal | left | mts of squamous cell lung carcinoma | no |

Notes: KPS - Karnofsky Performance Scale; FGS - Fluorescence-Guided Surgery with 5-aminolevulinic acid; mts – metastases.

**Supplementary Table S3.** Characteristics of patients with brain metastases.
