# Supplementary material for: Membrane-bound Heat Shock Protein mHsp70 Is Required for Migration and Invasion of Brain Tumors
Source: Cancer Res Commun. 2024 Aug 12;4(8):2025–44. doi: 10.1158/2767-9764.CRC-24-0094 (PMC11317918; doi:10.1158/2767-9764.CRC-24-0094)
Supplement: Supplementary Table S4 — Grade content and co-localization of biomarkers (Hsp70, Nestin, SOX2) on histological preparations of human GBM. [file crc-24-0094_supplementary_table_s4_suppst4.docx]

| **Zone** | **Presence of marker, %** | | | **Absence of marker, %** | **Colocalization, %** | |
| --- | --- | --- | --- | --- | --- | --- |
|  | **Hsp70** | **Nestin** | **SOX2** |  | **Hsp70/Nestin** | **Hsp70/SOX2** |
| **VT** | 51 | 16 | 9 | 24 | 67 | 35 |
| **NA** | 37 | 17 | 0 | 46 | 20 | 0 |

Notes: VT - viable tissue; NA - necrosis area; the number of cells on the image (with and without markers) was taken as 100%.

**Supplementary Table S4.** Grade content and co-localization of biomarkers (Hsp70, Nestin, SOX2) on histological preparations of human GBM.
