# Supplementary material for: Membrane-bound Heat Shock Protein mHsp70 Is Required for Migration and Invasion of Brain Tumors
Source: Cancer Res Commun. 2024 Aug 12;4(8):2025–44. doi: 10.1158/2767-9764.CRC-24-0094 (PMC11317918; doi:10.1158/2767-9764.CRC-24-0094)
Supplement: Supplementary Table S5 — List of proteins detected by mass spectrometric analysis of lipid rafts isolated from normal brain tissue (obtained from a patient with epilepsy). [file crc-24-0094_supplementary_table_s5_suppst5.docx]

| Uniprot Accession # | Name | -10LgP | Coverage(%) | Average Mass |
| --- | --- | --- | --- | --- |
| P68871 | HBB_HUMAN | 656,4474 | 29,93 | 15998,403 |
| P04216 | THY1_HUMAN | 431,6352 | 14,91 | 17934,717 |
| P21796 | VDAC1_HUMAN | 526,65216 | 14,84 | 30772,59 |
| P63261 | ACTG_HUMAN | 674,3588 | 11,73 | 41792,836 |
| P60201 | MYPR_HUMAN | 551,6562 | 11,55 | 30077,164 |
| P04406 | G3P_HUMAN | 614,1769 | 11,34 | 36053,207 |
| P99999 | CYC_HUMAN | 299,6581 | 10,48 | 11748,72 |
| P69905 | HBA_HUMAN | 657,8099 | 8,45 | 15257,544 |
| P02042 | HBD_HUMAN | 595,90985 | 8,16 | 16055,474 |
| P21579 | SYT1_HUMAN | 519,22394 | 7,58 | 47573,1 |
| P68366 | TBA4A_HUMAN | 624,2849 | 4,46 | 49924,4 |
| Q9BQE3 | TBA1C_HUMAN | 621,6736 | 4,45 | 49895,324 |
| Q9NY65 | TBA8_HUMAN | 575,44104 | 4,45 | 50093,543 |
| Q6PEY2 | TBA3E_HUMAN | 602,4744 | 4,44 | 49858,54 |
| P68363 | TBA1B_HUMAN | 642,21796 | 4,43 | 50151,63 |
| Q71U36 | TBA1A_HUMAN | 643,208 | 4,43 | 50135,63 |
| Q99623 | PHB2_HUMAN | 499,99423 | 4,35 | 33296,38 |
| P62873 | GBB1_HUMAN | 481,03625 | 3,24 | 37376,96 |
| Q9HAV0 | GBB4_HUMAN | 424,88116 | 3,24 | 37567,18 |
| P62879 | GBB2_HUMAN | 444,1426 | 3,24 | 37331,027 |
| P60174 | TPIS_HUMAN | 442,31604 | 3,21 | 26669,484 |
| P11488 | GNAT1_HUMAN | 286,7458 | 3,14 | 40040,785 |
| A8MTJ3 | GNAT3_HUMAN | 304,9811 | 3,11 | 40356,996 |
| P08754 | GNAI3_HUMAN | 467,9624 | 3,11 | 40532,188 |
| P09471 | GNAO_HUMAN | 497,62323 | 3,11 | 40050,562 |
| P63096 | GNAI1_HUMAN | 480,89413 | 3,11 | 40361,08 |
| P04899 | GNAI2_HUMAN | 513,2474 | 3,1 | 40450,902 |
| P00403 | COX2_HUMAN | 356,39072 | 3,08 | 25565,016 |
| Q14344 | GNA13_HUMAN | 465,20352 | 2,92 | 44049,57 |
| Q03113 | GNA12_HUMAN | 387,6542 | 2,89 | 44279,227 |
| Q8N9I0 | SYT2_HUMAN | 456,74054 | 2,86 | 46872,22 |
| P63092 | GNAS2_HUMAN | 449,7973 | 2,79 | 45664,58 |
| P13637 | AT1A3_HUMAN | 662,1504 | 2,67 | 111748,54 |
| P50993 | AT1A2_HUMAN | 656,9249 | 2,65 | 112265,44 |
| P05023 | AT1A1_HUMAN | 660,5724 | 2,64 | 112896,09 |
| Q13885 | TBB2A_HUMAN | 601,3033 | 2,25 | 49906,97 |
| Q9BVA1 | TBB2B_HUMAN | 601,27234 | 2,25 | 49953,055 |
| P04350 | TBB4A_HUMAN | 609,56476 | 2,25 | 49585,766 |
| P68371 | TBB4B_HUMAN | 608,953 | 2,25 | 49831 |
| P07437 | TBB5_HUMAN | 601,17285 | 2,25 | 49670,812 |
| Q13509 | TBB3_HUMAN | 570,62976 | 2,22 | 50432,676 |
| Q6S8J3 | POTEE_HUMAN | 565,98395 | 2,14 | 121363,43 |
| P02768 | ALBU_HUMAN | 729,6823 | 1,48 | 69366,68 |
| Q5JWF2 | GNAS1_HUMAN | 452,85657 | 1,06 | 111024,516 |
| P35579 | MYH9_HUMAN | 749,2537 | 0,92 | 226532,23 |
| P54707 | AT12A_HUMAN | 438,87643 | 0,87 | 115510,52 |
| Q13733 | AT1A4_HUMAN | 510,0173 | 0,87 | 114166,46 |
| P04114 | APOB_HUMAN | 812,08795 | 0,44 | 515604,72 |
| P13611 | CSPG2_HUMAN | 627,53076 | 0,35 | 372820,03 |
| Q8WZ42 | TITIN_HUMAN | 413,6105 | 0,02 | 3816030 |

**Supplementary Table S5.** List of proteins detected by mass spectrometric analysis of lipid rafts isolated from normal brain tissue (obtained from a patient with epilepsy).
