# Supplementary figures and images for: Membrane-bound Heat Shock Protein mHsp70 Is Required for Migration and Invasion of Brain Tumors
Source: Cancer Res Commun. 2024 Aug 12;4(8):2025–44. doi: 10.1158/2767-9764.CRC-24-0094 (PMC11317918; doi:10.1158/2767-9764.CRC-24-0094)

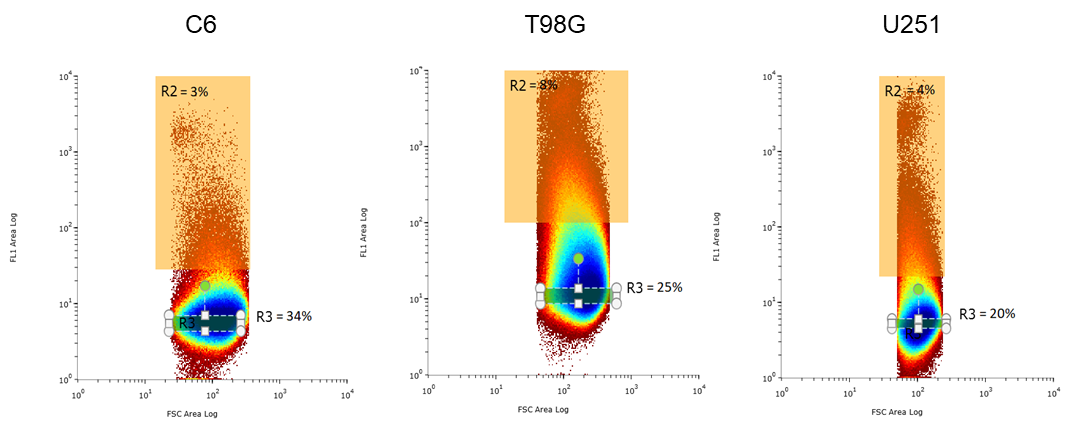


**Supplementary Figure S1.** Gating of mHsp70High cells (field R2) and mHsp70Low cells (field R3) by FACS.

Supplement: Supplementary Figure S1 — Gating of mHsp70High cells (field R2) and mHsp70Low cells (field R3) by FACS. [file crc-24-0094_supplementary_figure_s1_supps1.docx]

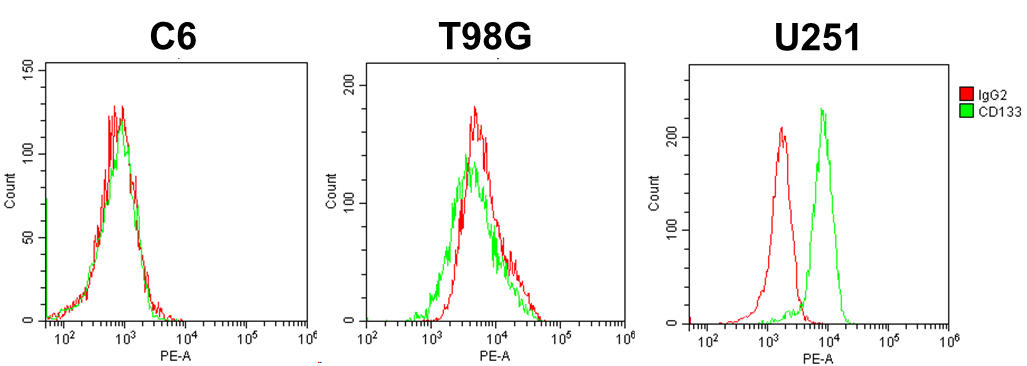


**Supplementary Figure S2.** CD133 expression in C6, T98G and U251 cell lines measured by flow cytometry.

Supplement: Supplementary Figure S2 — CD133 expression in C6, T98G and U251 cell lines measured by flow cytometry. [file crc-24-0094_supplementary_figure_s2_supps2.docx]
