# Supplementary material for: Membrane-bound Heat Shock Protein mHsp70 Is Required for Migration and Invasion of Brain Tumors
Source: Cancer Res Commun. 2024 Aug 12;4(8):2025–44. doi: 10.1158/2767-9764.CRC-24-0094 (PMC11317918; doi:10.1158/2767-9764.CRC-24-0094)
Supplement: Supplementary Figure S3 — Preparing a tumor sample for confocal microscopy. [file crc-24-0094_supplementary_figure_s3_supps3.docx]

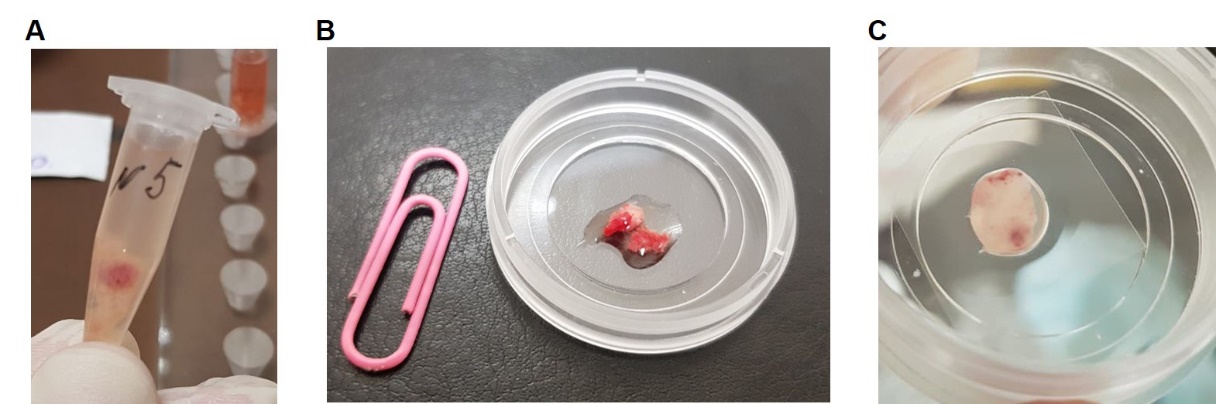


**Supplementary Figure S3.** Preparing a tumor sample for confocal microscopy. (A) After surgery, the material is delivered in a transport medium. (B) After antibody staining and washing, the sample is placed on the glass bottom of a microscopy dish. (С) The sample is covered with a coverslip and ready for microscopy.
