# Supplementary material for: Membrane-bound Heat Shock Protein mHsp70 Is Required for Migration and Invasion of Brain Tumors
Source: Cancer Res Commun. 2024 Aug 12;4(8):2025–44. doi: 10.1158/2767-9764.CRC-24-0094 (PMC11317918; doi:10.1158/2767-9764.CRC-24-0094)
Supplement: Supplementary Figure S4 — The sequence of constructing a phenotyping map for histological sections of human GBM. [file crc-24-0094_supplementary_figure_s4_supps4.docx]

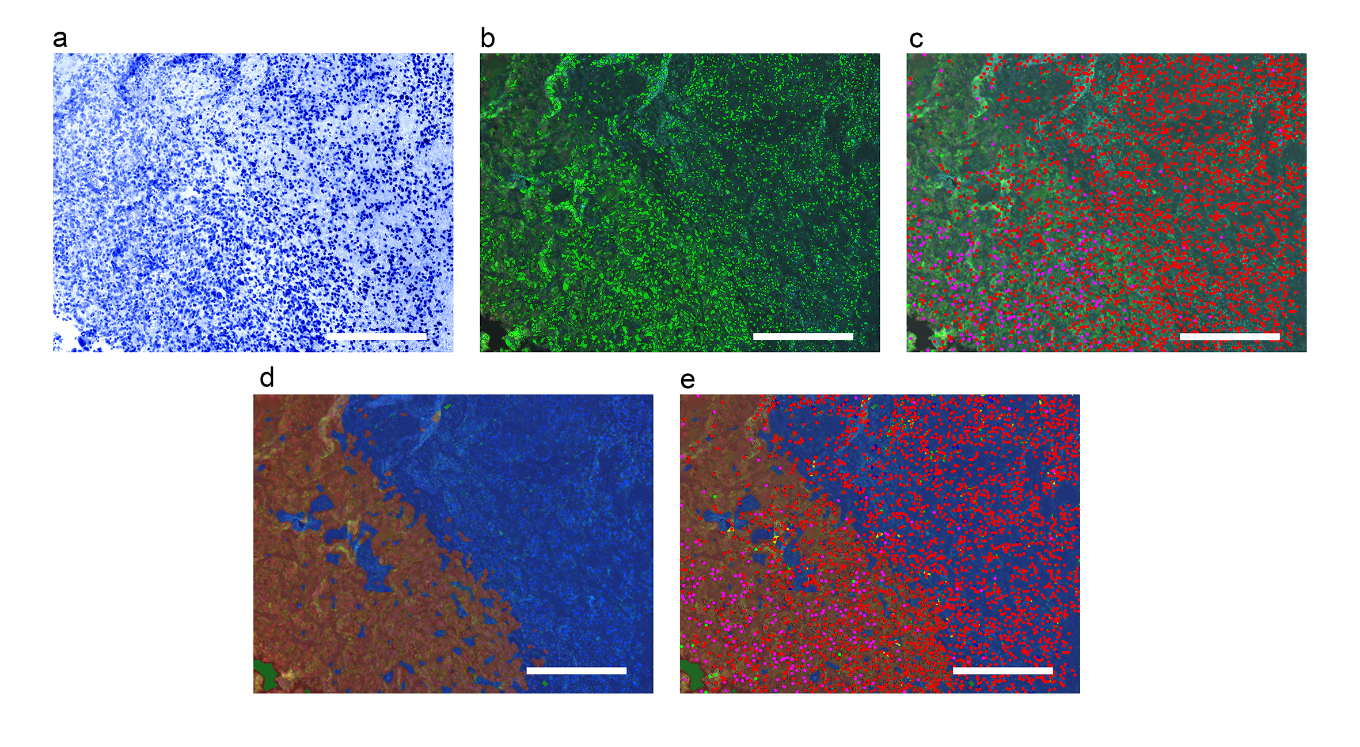


**Supplementary Figure S4.** The sequence of constructing a phenotyping map for histological sections of human GBM. (a) Staining of DAPI nuclei, (b) nuclear phenotyping (green), (c) cell phenotyping by the Hsp70, Nestin, and SOX2 markers: red, magenta, and green respectively, (d) segmentation of tissue into a zone of necrosis (blue) and viable (VT) tissue (brown), (e) Map of phenotyping by tissue zones (necrosis and VT) and cells containing Hsp70, Nestin, and SOX2. Scale bar, 400 μm.
