# Supplementary material for: Membrane-bound Heat Shock Protein mHsp70 Is Required for Migration and Invasion of Brain Tumors
Source: Cancer Res Commun. 2024 Aug 12;4(8):2025–44. doi: 10.1158/2767-9764.CRC-24-0094 (PMC11317918; doi:10.1158/2767-9764.CRC-24-0094)
Supplement: Supplementary Figure S5 — Evaluation of biomarkers co-localization. [file crc-24-0094_supplementary_figure_s5_supps5.docx]

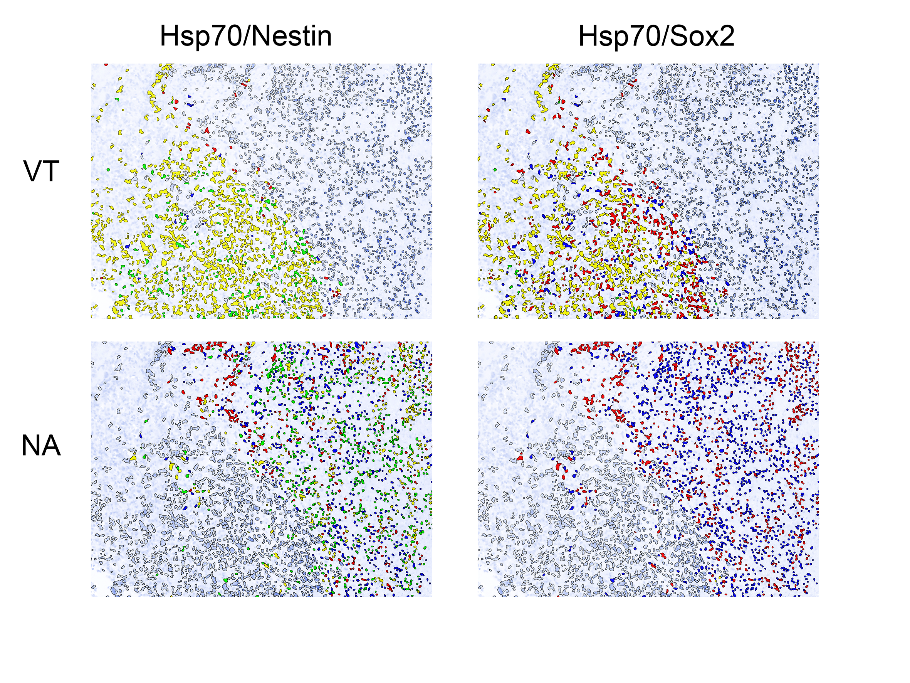


**Supplementary Figure S5.** Evaluation of biomarkers co-localization. Hsp70 (red color); Nestin and SOX2 (green color); co-localization of Hsp70 with Nestin and SOX2 (yellow color); no markers (blue color). Top row is a zone of viable tissue (VT); Bottom row is a necrosis area (NA).
