# Supplementary material for: Membrane-bound Heat Shock Protein mHsp70 Is Required for Migration and Invasion of Brain Tumors
Source: Cancer Res Commun. 2024 Aug 12;4(8):2025–44. doi: 10.1158/2767-9764.CRC-24-0094 (PMC11317918; doi:10.1158/2767-9764.CRC-24-0094)
Supplement: Supplementary Figure S6 — Motility of Hsp70-positive cells in a tumor sample. [file crc-24-0094_supplementary_figure_s6_supps6.docx]

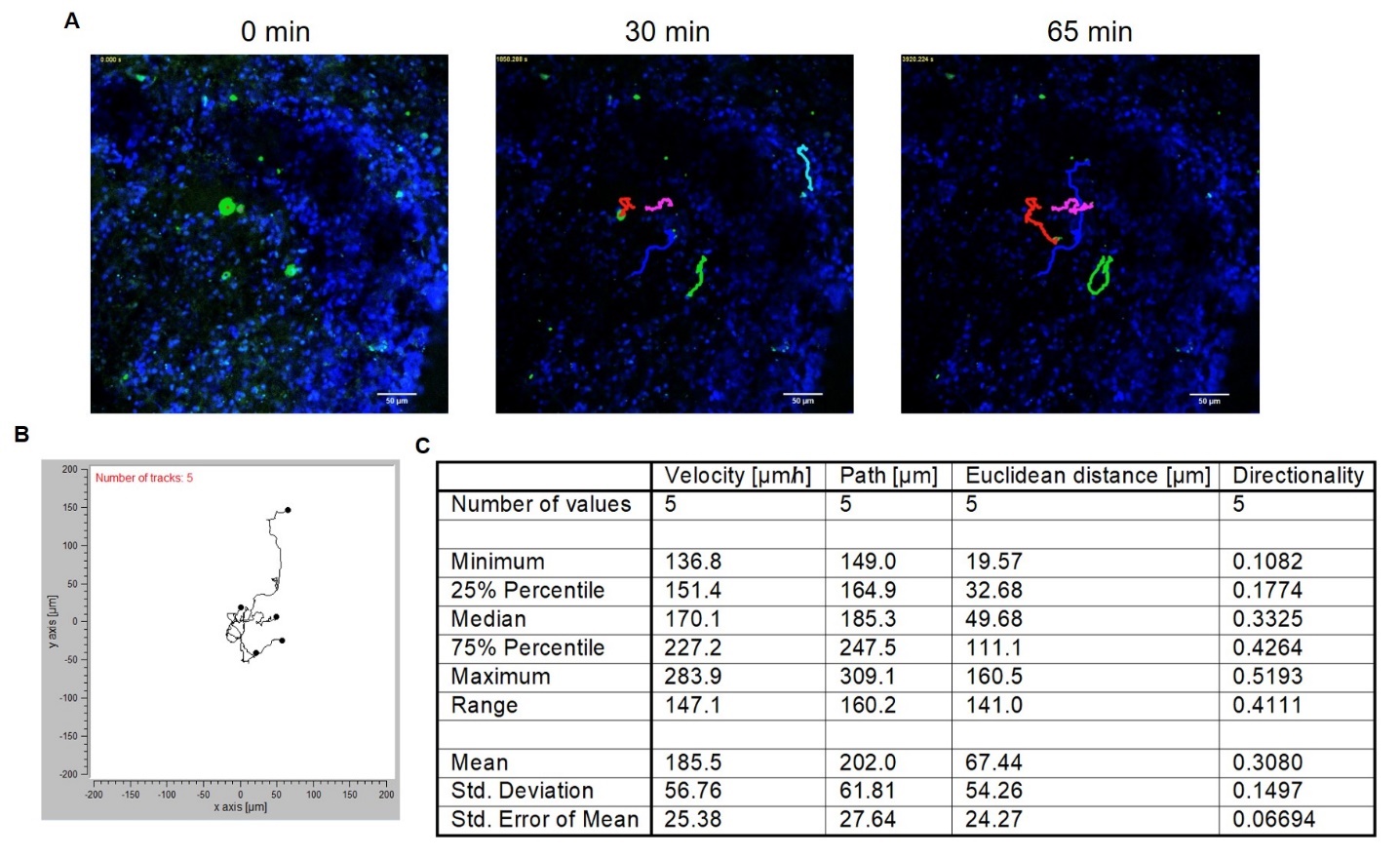


**Supplementary Figure S6.** Motility of Hsp70-positive cells in a tumor sample. Motility was detected using laser scanning confocal microscopy. (A) Examples of three images showing the field of view at the beginning, middle and end of the observation period. Images were combined from two channels: blue – stained cell nuclei, green - staining for mHsp70. Colored lines indicate tracks of moving cells. (B) Normalized tracks collected from confocal images. (C) Descriptive statistics for cell motility measurements performed using intravital laser scanning confocal microscopy of glioblastoma specimen obtained from GBM patients. Motile Hsp70-positive cells were tracked using Manual Tracking plugin in ImageJ software.
