# Supplementary material for: Membrane-bound Heat Shock Protein mHsp70 Is Required for Migration and Invasion of Brain Tumors
Source: Cancer Res Commun. 2024 Aug 12;4(8):2025–44. doi: 10.1158/2767-9764.CRC-24-0094 (PMC11317918; doi:10.1158/2767-9764.CRC-24-0094)
Supplement: Supplementary Figure S7 — Analysis of the mass-spectrometry data from isolated lipid rafts. [file crc-24-0094_supplementary_figure_s7_supps7.docx]

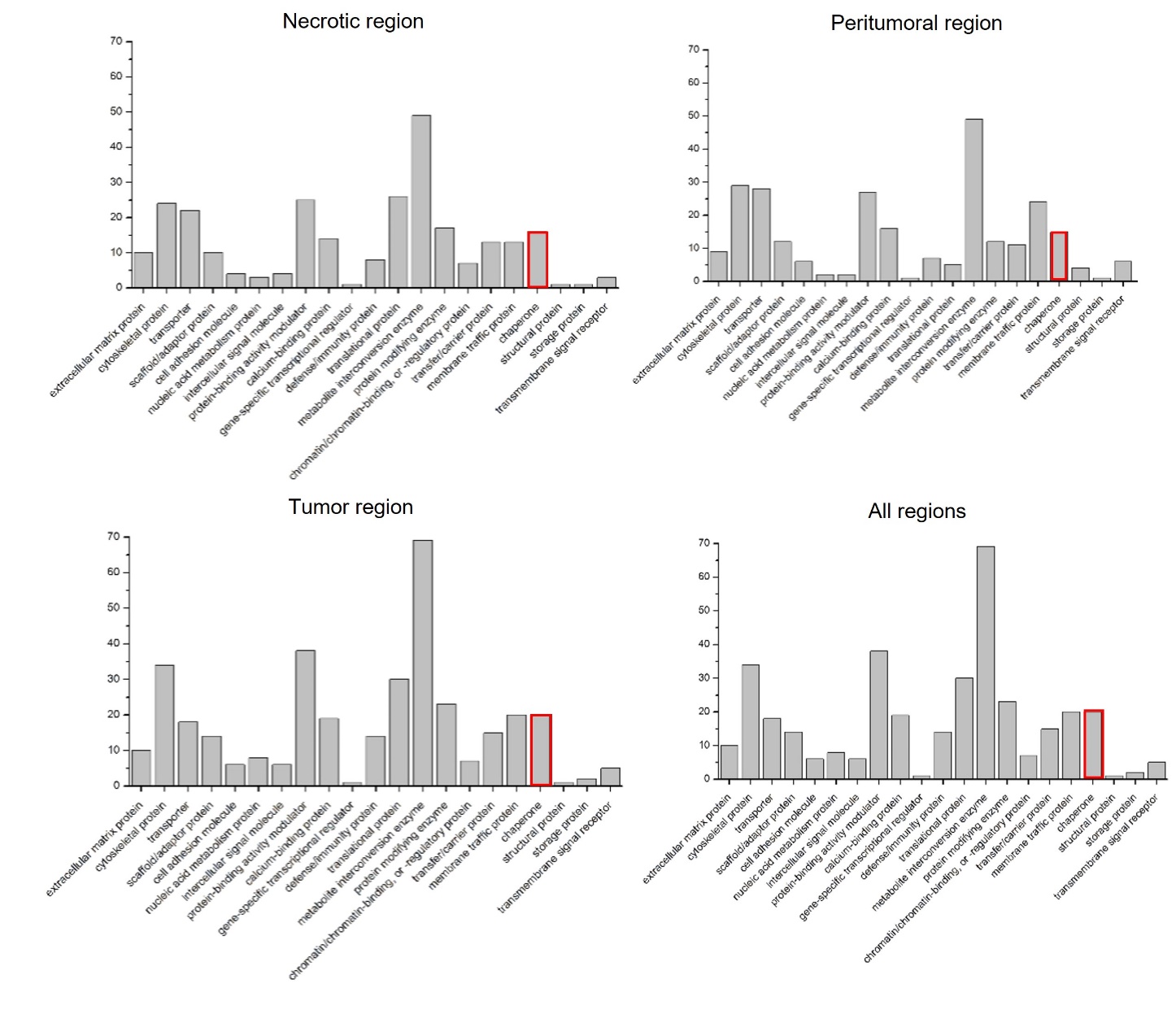


**Supplementary Figure S7.** Analysis of the mass-spectrometry data from isolated lipid rafts. Protein functional groups identified using the STRING database in the proteome of lipid rafts from three tumor zones.
