# Supplementary material for: Membrane-bound Heat Shock Protein mHsp70 Is Required for Migration and Invasion of Brain Tumors
Source: Cancer Res Commun. 2024 Aug 12;4(8):2025–44. doi: 10.1158/2767-9764.CRC-24-0094 (PMC11317918; doi:10.1158/2767-9764.CRC-24-0094)
Supplement: Supplementary Figure S8 — Effect of Hsp70 inhibitors PES and JG-98 on cell viability analyzed using the MTT assay. [file crc-24-0094_supplementary_figure_s8_supps8.docx]

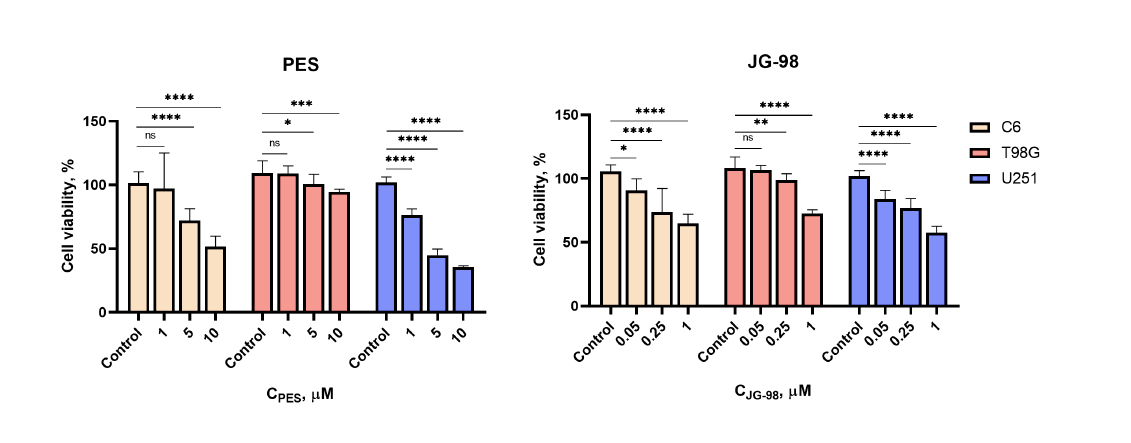


**Supplementary Figure S8.** Effect of Hsp70 inhibitors PES and JG-98 on cell viability analyzed using the MTT assay. The cell viability was averaged from 2-3 experiments for each cell line (median with 95% CI).
