# Supplementary material for: Membrane-bound Heat Shock Protein mHsp70 Is Required for Migration and Invasion of Brain Tumors
Source: Cancer Res Commun. 2024 Aug 12;4(8):2025–44. doi: 10.1158/2767-9764.CRC-24-0094 (PMC11317918; doi:10.1158/2767-9764.CRC-24-0094)
Supplement: Supplementary Figure S9 — Confocal microscopy images of primary glioblastoma cells stained for mHsp70 and SOX2. [file crc-24-0094_supplementary_figure_s9_supps9.docx]

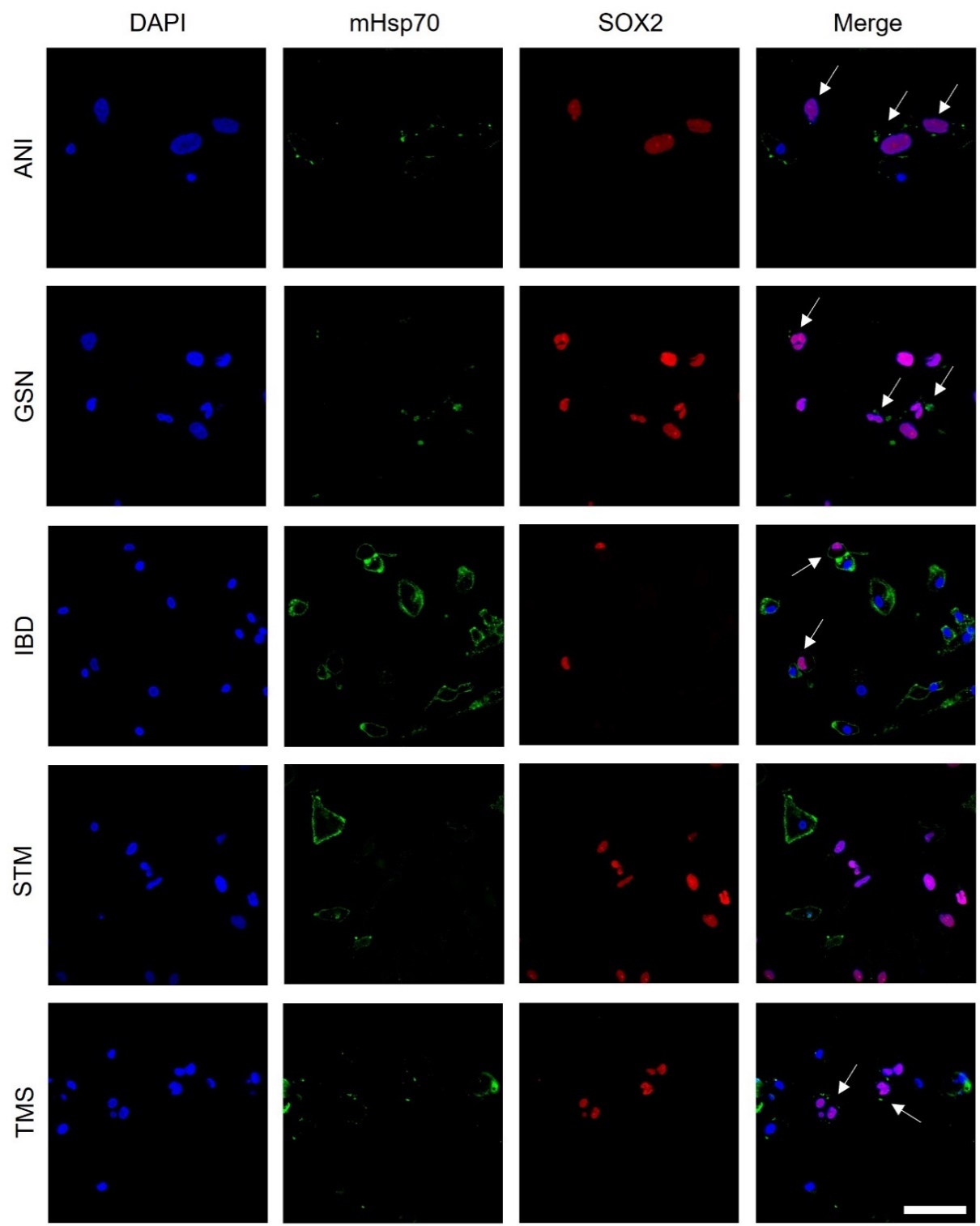


**Supplementary Figure S9.** Confocal microscopy images of primary glioblastoma cells stained for mHsp70 and SOX2. Samples from patients (n = 5) ANI, GSN, IBD, STM, and TMS were stained for mHsp70 and neural stem cell marker SOX2. DAPI was applied for nucleus staining (blue). FITC-Hsp70mAb was used for detecting mHsp70 on plasma membrane (green). eFluor™ 570-SOX2 was used for detecting SOX2 protein. Scale bars, 50 μm.
