# Supplementary material for: Membrane-bound Heat Shock Protein mHsp70 Is Required for Migration and Invasion of Brain Tumors
Source: Cancer Res Commun. 2024 Aug 12;4(8):2025–44. doi: 10.1158/2767-9764.CRC-24-0094 (PMC11317918; doi:10.1158/2767-9764.CRC-24-0094)
Supplement: Supplementary Figure S10 — Confocal microscopy images of primary glioblastoma cells from patients stained for mHsp70 and Nestin. [file crc-24-0094_supplementary_figure_s10_supps10.docx]

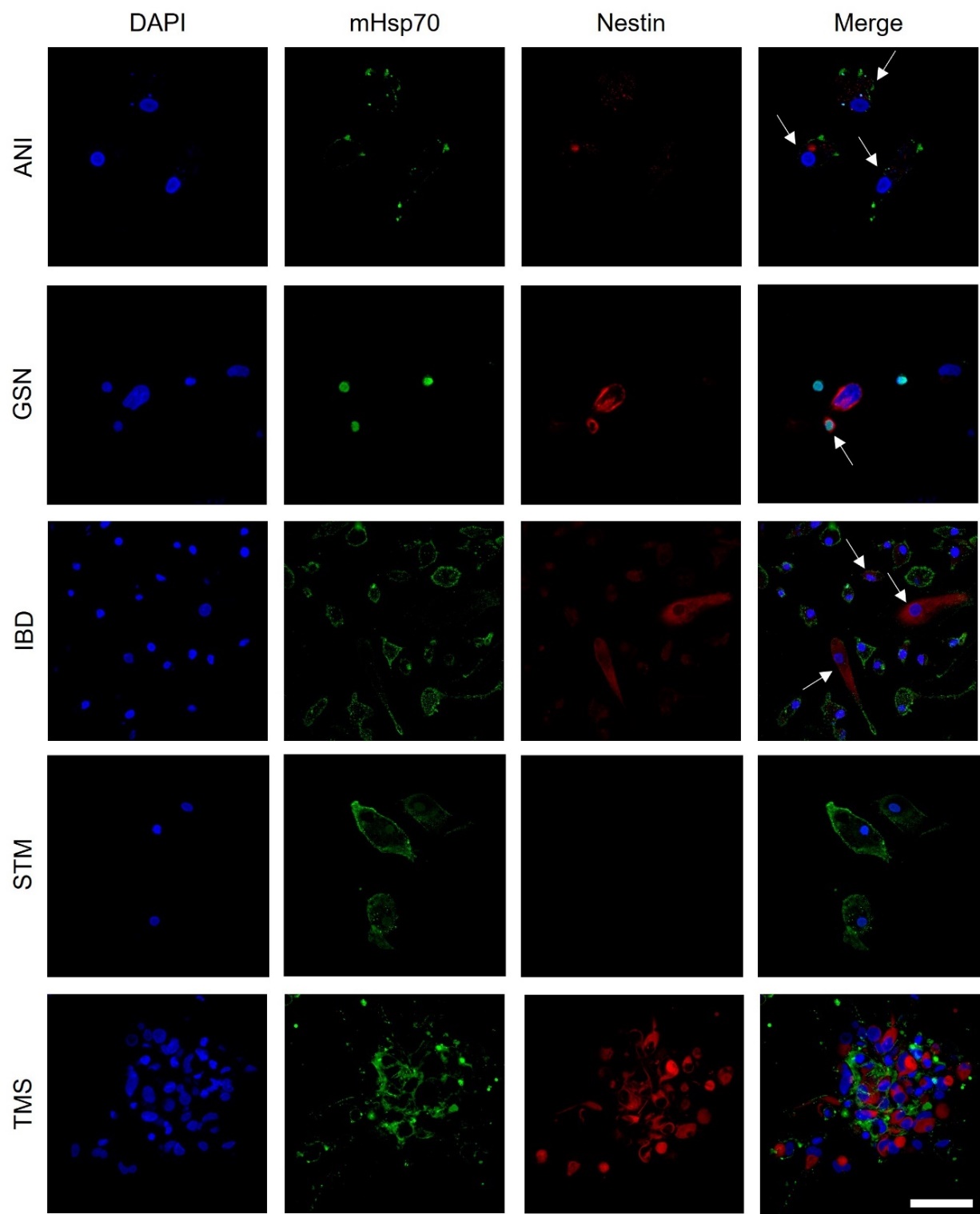


**Supplementary Figure S10.** Confocal microscopy images of primary glioblastoma cells from patients stained for mHsp70 and Nestin. Samples from patients (n = 5) ANI, GSN, IBD, STM, and TMS were stained for mHsp70 and neural stem cell marker Nestin. DAPI was applied for nucleus staining (blue). FITC-Hsp70mAb was used for detecting mHsp70 on plasma membrane (green). Alexa Fluor® 647-Nestin was used for detecting Nestin protein. Scale bars, 50 μm.
