# Supplementary material for: Membrane-bound Heat Shock Protein mHsp70 Is Required for Migration and Invasion of Brain Tumors
Source: Cancer Res Commun. 2024 Aug 12;4(8):2025–44. doi: 10.1158/2767-9764.CRC-24-0094 (PMC11317918; doi:10.1158/2767-9764.CRC-24-0094)
Supplement: Supplementary Figure S11 — Comparison of mean speed and track straightness on various matrices (PL, MG, FN) of patient-derived (non-sorted into high- and low-speed subpopulations) brain tumor cells treated with 1 µM PES or 50 nM JG-98. [file crc-24-0094_supplementary_figure_s11_supps11.docx]

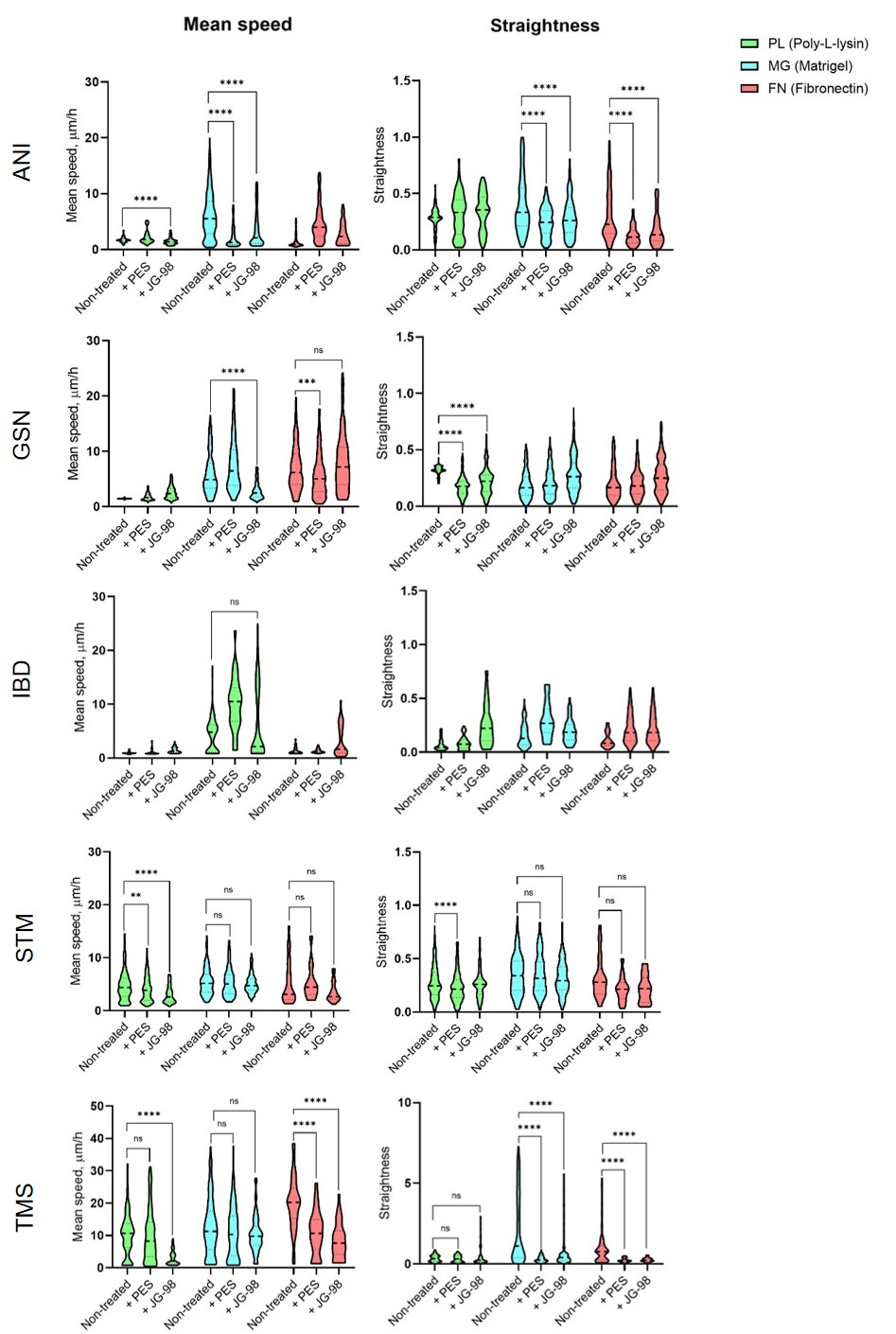


**Supplementary Figure S11.** Comparison of mean speed and track straightness on various matrices (PL, MG, FN) of patient-derived (non-sorted into high- and low-speed subpopulations) brain tumor cells treated with 1 µM PES or 50 nM JG-98 (n_tracks_ ≥ 294). Non-treated cells were used as a control. Data is presented as median ± 95% CI. Significant differences identified by the Wilcoxon test are shown as *p < 0.05, ** p < 0.01, ***p < 0.001, ****p < 0.0001, ns – not significant.
